# Microbial metabolite restricts 5-fluorouracil-resistant colonic tumor progression by sensitizing drug transporters via regulation of FOXO3-FOXM1 axis

**DOI:** 10.7150/thno.70754

**Published:** 2022-07-18

**Authors:** Sweta Ghosh, Rajbir Singh, Zachary Matthew Vanwinkle, Haixun Guo, Praveen Kumar Vemula, Ajay Goel, Bodduluri Haribabu, Venkatakrishna Rao Jala

**Affiliations:** 1 Department of Microbiology and Immunology, Brown Cancer Center, Center for Microbiomics, Inflammation and Pathogenicity, University of Louisville, Louisville, KY, USA.; 2 Department of Radiology, Center for Predictive Medicine, University of Louisville, Louisville, KY, USA.; 3 Institute for Stem Cell Biology and Regenerative Medicine (inStem), GKVK campus, Bangalore, Karnataka 560065, India.; 4 Department of Molecular Diagnostics and Experimental Therapeutics, City of Hope Comprehensive Cancer Center, Duarte, CA, USA.

**Keywords:** Chemoresistant colon cancer, 5-Fluorouracil, Microbial metabolite, Urolithin A, Chemosensitization, drug transporters, FOXO3-FOXM1 axis

## Abstract

The survival rate of colorectal cancer patients is adversely affected by the selection of tumors resistant to conventional anti-cancer drugs such as 5-fluorouracil (5FU). Although there is mounting evidence that commensal gut microbiota is essential for effective colon cancer treatment, the detailed molecular mechanisms and the role of gut microbial metabolites remain elusive. The goal of this study is to decipher the impact and mechanisms of gut microbial metabolite, urolithin A (UroA) and its structural analogue, UAS03 on reversal of 5FU-resistant (5FUR) colon cancers.

**Methods:** We have utilized the SW480 and HCT-116 parental (5FU-sensitive) and 5FUR colon cancer cells to examine the chemosensitization effects of UroA or UAS03 by using both *in vitro* and *in vivo* models. The effects of mono (UroA/UAS03/5FU) and combinatorial therapy (UroA/UAS03 + 5FU) on cell proliferation, apoptosis, cell migration and invasion, regulation of epithelial mesenchymal transition (EMT) mediators, expression and activities of drug transporters, and their regulatory transcription factors were examined using molecular, cellular, immunological and flowcytometric methods. Further, the anti-tumor effects of mono/combination therapy (UroA or UAS03 or 5FU or UroA/UAS03 + 5FU) were examined using pre-clinical models of 5FUR-tumor xenografts in NRGS mice and azoxymethane (AOM)-dextran sodium sulfate (DSS)-induced colon tumors.

**Results:** Our data showed that UroA or UAS03 in combination with 5FU significantly inhibited cell viability, proliferation, invasiveness as well as induced apoptosis of the 5FUR colon cancer cells compared to mono treatments. Mechanistically, UroA or UAS03 chemosensitized the 5FUR cancer cells by downregulating the expression and activities of drug transporters (MDR1, BCRP, MRP2 and MRP7) leading to a decrease in the efflux of 5FU. Further, our data suggested the UroA or UAS03 chemosensitized 5FUR cancer cells to 5FU treatment through regulating FOXO3-FOXM1 axis. Oral treatment with UroA or UAS03 in combination with low dose i.p. 5FU significantly reduced the growth of 5FUR-tumor xenografts in NRGS mice. Further, combination therapy significantly abrogated colonic tumors in AOM-DSS-induced colon tumors in mice.

**Conclusions:** In summary, gut microbial metabolite UroA and its structural analogue UAS03 chemosensitized the 5FUR colon cancers for effective 5FU chemotherapy. This study provided the novel characteristics of gut microbial metabolites to have significant translational implications in drug-resistant cancer therapeutics.

## Introduction

Colorectal cancer (CRC) is the third leading cause of cancer-related deaths in the United States with an overall lifetime risk of developing CRC of 4.3% for men and 4.0% for women 1. The relative 5-year survival is about 64.7% for CRC patients. Current treatment strategies for CRC include surgery (early stages of cancer), radiation therapy, chemotherapy and immune therapy. Despite improved chemotherapeutic strategies for CRC treatment, resistance to chemotherapy agents remains a major obstacle. 5-fluorouarcil (5FU) is a first-line chemotherapeutic drug used to treat several cancers including colon cancer. However, the response rate for 5FU treatment of CRC is only 10-15% 2. In most cases, the tumors that do respond to 5FU treatment eventually develop chemoresistance. The underlying molecular mechanisms involved in chemoresistance are not fully understood and the approaches to overcome chemoresistance have not been identified 3.

Numerous studies have highlighted the importance of gut microbiota in the regulation of colon cancers 4-7. Several factors including infection, diet, use of antibiotics, and surgery, as well as host genetics are known to affect the dynamics of microbial communities in the intestine and play a critical role in modulating both the innate and adaptive immune systems and the development of various immunity-related diseases 8-15. Adverse effects of microbial dysbiosis may not be immediately apparent, but the long-term consequences of microbial dysbiosis on human health include obesity, metabolic disorders, inflammatory bowel diseases and neurological disorders, all of which contribute to increased risk of colon cancer 16-18. This study focuses on investigating the effects of gut microbial metabolites on the process of chemosensitization to anti-cancer therapeutics such as 5FU.

Dietary polyphenols are responsible for some of the beneficial health effects associated with high consumption of fruits and vegetables in humans. Among these polyphenols, ellagitannins and ellagic acid (EA) are major components of berries and pomegranates and exert anti-inflammatory, anti-oxidant and anti-carcinogenic effects 19. Because the intestinal absorption (bioavailability) of ellagitannins and EA is poor, it has been suggested that the potential health benefits rendered by these compounds are due to microbial catabolism of these compounds to urolithins 20. Among urolithins, urolithin A (3,8-dihydroxybenzo[c]chromen-6-one, UroA) exhibits a high rate of intestinal absorption 21 and exerts anti-inflammatory, anti-oxidative, and anti-ageing activities 22-25. UroA production is not uniform among individuals who consume comparable amounts of EA/ET-rich foods due to the presence/absence and varied levels of UroA-producing bacteria in each individual 26. Independent studies in healthy volunteers suggested that only 40-50% of humans were capable of producing UroA following pomegranate juice or walnut consumption 27-37. Andreux et al 38 showed that oral consumption of UroA in a human phase 1 study of healthy or sedentary elders either as a single dose or as multiple doses (500 mg or 1000 mg daily) over a 4-weeks is not toxic. A recent study in humans also highlighted that the direct consumption of UroA supplementation could circumvent the requirement of UroA-producing bacteria and EA/ET-rich diets 37

Previously, we have demonstrated that oral treatment with UroA mitigated chemically-induced colitis in mice 39. We showed that oral treatment with UroA enhanced gut barrier function through upregulation of intestinal epithelial tight junction proteins leading to decreased gut permeability and inflammation. A potential disadvantage of oral consumption of UroA as a potential health-promoting nutraceutical or as a potential pharmaceutical is that UroA is rapidly hydrolyzed by gastric hydrolases at low pH 39. We therefore developed a novel synthetic UroA analogue, UAS03, which exhibited better stability at low pH and better therapeutic efficacy at low doses in murine peritonitis and colitis models compared to UroA 39. Further, we showed that UroA and UAS03 mediated gut barrier protective activities are dependent on the aryl hydrocarbon receptor (AhR) and the nuclear factor erythroid 2-related factor 2 (Nrf2) 39. Because Nrf2 has been associated with resistance to chemotherapeutic agents due to its ability to downregulate the expression of multi drug-resistance-associated transporters 40-42, we reasoned that UroA and UAS03 could potentially enhance the susceptibility of 5FU-resistant cells to 5FU. In the current study, we examined whether UroA and UAS03 chemosensitize 5FUR colon cancer to 5FU treatment using both *in vitro* and *in vivo* models. We report that both UroA and UAS03 chemosensitize 5FUR colon cancer by down-regulating the expression of drug transporters via the FOXO3-FOXM1 signalling pathway. Our results show that UroA/UAS03 may be efficacious in conjunction with 5FU in treating 5FU-resistant colon cancer, suggesting a potential role of gut microbial metabolites to improve the cancer patients' outcome.

## Methods

**Cell culture:** Colon carcinoma cell lines, HCT-116 and SW-480 cells and their 5fluorouracil (5-FU; Sigma Aldrich Cat. no F6627) resistant counterparts (HCT-FUR and SW480-FUR) were obtained from Dr. Ajay Goel, City of Hope Comprehensive Cancer Center, Duarte, CA, USA. The 5FU-senstive (parental) cells were maintained in Iscove's medium supplemented with 10% fetal bovine serum (FBS) and 1X antibiotics (100 U/mL penicillin, and 100 µg/mL streptomycin; Sigma Aldrich Cat no P4333) at 37 ºC in a humified incubator with 5% CO_2_. The resistant cells were grown/maintained in Iscove's complete Medium (10% FBS and 1X antibiotics) supplemented with 5 µM 5-FU.

**Mice:** NRGS (NOD.Cg-*Rag1^tm1Mom^ Il2rg^tm1Wjl^* Tg(CMV-IL3,CSF2,KITLG)1Eav/J ) and C57BL/6 wild type (WT) breeding pairs mice were obtained from Jackson Laboratories. All the experimental animals were generated at U of L specific pathogen-free (SPF) animal facility at the research resources center of the University of Louisville. All animals were housed in ventilated cages under controlled conditions of light and fed ad libitum in a barrier facility under specific pathogen free (SPF) conditions. The Institutional Animal Care and Use Committee at the University of Louisville approved all of the experimental protocols.

**Cell viability assay:** For assaying cell viability, cells (5 X 10^3^ cells/well) were seeded in 96 well plates and incubated with 5-FU (0, 3.12, 6.25, 12.5, 25, 50 and 100 µM) with or without UroA/UAS03 (indicated doses) for 24, 48 and 72 h. Each treatment was tested in four replicate wells. Cells treated with vehicle (0.1% DMSO) were used as negative controls, whereas the wells without cells were used as blank control. Cell viability was determined using 3-(4,5-Dimethylthiazol-2-yl)-2,5-Diphenyltetrazolium Bromide (MTT) cytotoxicity. Briefly, after incubation with drugs, MTT (20 µL; 5 mg/mL) was added to each well and incubated for 4 h. After 4 h, the medium with MTT solution was removed and 100 µL of dimethylsulfoxide (DMSO) was added and incubated for 15 min at room temperature on a shaker. The absorbance of formazan crystals (generated upon mitochondrial-mediated reduction of MTT) was recorded at 570 nm using a Synergy HT Microplate Reader (Biotek, VT, USA).

**Western blot analysis:** Parental or 5FUR colon cancer cells were treated with 5-FU (10 and 50 µM) with or without UroA (10 or 50 µM) and UAS03 (10 or 50 µM) for 24, 48 or 72 h. Cells were lysed using RIPA buffer containing 1X protease inhibitors (Sigma Aldrich). The protein concentration was determined by BCA protein estimation kit (Thermo Scientific) with bovine serum albumin (BSA) as a standard. The protein samples (25 μg) were separated on NuPAGE^TM^ 4-12% Bis-Tris gel (Novex Life technologies) and then transferred onto PVDF membrane (0.22 µm pore; Millipore, USA). The membranes were blocked at room temperature for 1 h with blocking buffer (5% w/v skim milk in TBS buffer). Later, the membranes were probed with the indicated primary antibodies overnight at 4 ºC. After incubation, blots were washed and further incubated with respective species-specific secondary antibodies conjugated with horseradish peroxidase (HRP) (Santa Cruz Biotechnology, Santa Cruz, CA) for 1 h. β-actin or GAPDH served as loading control. A list of specific antibodies, source, and dilutions used are provided in Table 1. Protein bands were detected using HRP chemiluminescent substrate (ImageQuant LAS 4000) and quantified using Image J software.

**RNA isolation:** Total RNA was isolated after indicated treatments using Maxwell® 16 LEV simplyRNA kit (Promega) and RNA was reverse transcribed using a Taqman^TM^ reverse transcription kit (Applied Biosystem, USA). The transcribed cDNA was diluted five-fold and mixed with specific gene primers (100 nM) and 1X SYBR green reaction mix (Power SYBR® Green PCR Master Mix; Applied Biosystems, USA). Alterations in gene expression levels were analyzed using a CFX96TM Real-Time System (Bio-Rad). Fold change in expression was calculated using the 2^-ΔΔCT^ method normalized with untreated control, and GAPDH or ß-actin were used as an internal control.

**Apoptosis assays**: For apoptosis assays, cells were treated with 5-FU (50 μM) in the absence or presence of UroA (50 μM) or UAS03 (50 μM) for 24 h. The cells (1 x 10^6^ cells/mL) were harvested and resuspended in 1X binding buffer and stained with Annexin V- FITC/ PI (Annexin V-FITC Apoptosis Detection Kit with PI, BD Biosciences, USA) according to the manufacturer's instructions. The percentages of apoptotic cells were determined by flow cytometry (BD FACSCanto, BD biosciences, USA). Data were analyzed using FlowJo v10 software.

**Efflux assay:** To evaluate the basal levels of the efflux transporters Pgp, BCRP and MRP2, we performed efflux assays using parental (5-FU sensitive) and 5-FU resistant (FUR) cells. Briefly, cells (0.1 x 10^6^/well) were grown to confluency in 12 well plates and treated with the Pgp or BCRP substrates Rhodamine 123 (Rh123, 5 µM) or mitoxantrone (MTX, 10 µM), respectively, for 90 min. Similarly, MRP2 functional activity was assessed by incubating cells (90 min) with 5(6)-carboxy-2,′7′-dichlorofluorescein diacetate (CDCFDA; 10 µM), which itself is not a substrate, but is hydrolyzed by intracellular esterases to form the MRP2 fluorescent substrate 5-(and-6)-carboxy-2',7'-dichlorofluorescein (CDFDA). After incubation, cells were washed with ice-cold PBS three times and lysed with 1% Triton X-100 in PBS. The lysate was then centrifuged at 10,000 rpm for 5 min at room temperature and supernatants were collected. Fluorescence was measured in lysates for Rh123 (Ex. 485 nm and Em 525 nm), MTX (Ex. 610 nm and Em 685 nm) or (CDFDA; Ex. 480 nm and Em 525 nm) for P-gp, BCRP and MRP2, respectively. To evaluate the effect of 5-FU treatment on the levels of the efflux transporters, FUR cells were treated with 5-FU (50 µM) in the presence or absence of UroA and UAS03 (10 and 50 µM) and efflux assays were carried out using the respective substrates as described above.

**Intracellular 5FU measurement:** Parental HCT-116 and HCT-5FUR cells (10,000 cells/well) were plated in 96-well plated and grown overnight. Cells were treated with vehicle (0.1% DMSO) or UroA (50 μM) or UAS03 (50 μM) for 24 h in quadruplicates (n = 4). After 24 h, cells were washed with 1X PBS (3 times) and treated with vehicle or 5FU (50 μM) for 90 min. At the end of treatment, the supernatant was discarded, and cells were washed three times with 0.5 ml of ice-cold 1X PBS. Then the cells in each well were mixed thoroughly with 100 µL of 10% MeOH, vortexed for 1 min, and 200 µL of methanol was added, vortexed for 1min, then centrifuged at 10,000 *g* for 10 min. The supernatant was collected into new tube and evaporated in a SpeedVac for 30 min at room temperature. The residue in each tube was dissolved in 300 µL of 1% ACN aqueous solution, vortexed for 1 min, and centrifuged at 10,000 *g* for 10 min. The supernatant was collected for HPLC analysis. ***Liquid Chromatographic Conditions***: Chromatographic separation for 5-FU was achieved using a method adapted from literature 43. Briefly, an Agilent 1120 compact LC system equipped with an Agilent Eclipse Plus C18 column (5 *μ*m, 4.6 × 150 mm) was used for analysis, with the temperature maintained at 25 °C. Hydrochloric acid (20 mM) in water (A) and ACN (B) was used as the mobile phase using the following elution gradient: 0-5 min, 1%-1% B; 5.01-6 min, 1% to 70% B; 6.01-7 min, 70% to 100% B; 7.01-8 min, 100%-100% B; 8.01-9 min, 100% to 1% B; 9-10 min, 1%-1%B. Chromatographic separation of the analyte was achieved within 5 min, with a total run time of 10 min. A flow rate of 1 ml/min and UV 270 nm were used for the analysis. A standard curve of 5FU was generated with three doses of 5FU (0.026, 0.26, and 2.6 µg), with R^2^ = 0.9987. 5FU (RT = 2.84 min) was successfully separated from the other two chemicals (UroA: RT = 9.22 min; UAS03: RT = 9.22 min) under similar conditions.

**Immunofluorescence and confocal microscopy imaging:** HCT-116 FUR cells were grown on 8-well chambered slides (Nunc® Lab-Tek™ II Chamber Slide™ System) and treated with vehicle or 5FU or UroA or UAS03 or 5FU+UroA or 5FU+UAS03 for 24 h. Cells were fixed with chilled methanol. Fixed cells were stained with either anti-E cadherin or β catenin antibodies (1:200 dilution) followed by Alexa flour 488-conjugated secondary antibody (1:500 dilution). The stained cells on slides were mounted with VECTASHIELD HardSet™ antifade mounting medium with DAPI (Vector Laboratories). Images were captured using Nikon A1R confocal microscope with appropriate laser channels. All the lists of antibodies and dilutions are provided in Table 1.

**Assessment of cell proliferation by Ki-67 staining**: For Ki67 staining, treated cells (vehicle (0.1% DMSO) or 5FU or UroA or UAS03 or 5FU+UroA or 5FU+UAS03) were centrifuged at 350 x g for 5 min and washed 3 times with 1X PBS. Cells were incubated in fixation/permeabilization buffer (Biolegend) on ice for 45 min. These cells were washed twice with cell staining buffer and incubated with fluorochrome-conjugated Ki67 antibody for 30 min at room temperature. Cells were washed and resuspended in 0.5 ml cell staining buffer for flow cytometric analysis. The acquisition of cells was performed using BD FACSCanto flow cytometer and the data analyzed using FlowJo v10 software.

**Scratch assay:** Cell migration was also assessed with standard wound healing or scratch assay as described 44. HCT-116-FUR or SW480-FUR cells (10^5^ cells/well) were plated in 12-well plate. At 80-90% confluency a scratch was made using 200 µl pipette tip. Cells were then treated with 5-FU (50 µM) in the presence or absence of UroA and UAS03 (50 µM). Cell migrated to wound or scratch area were evaluated at 24 h.

**Invasion assay**: For invasion assay, SW480-FUR and HCT-116 FUR cells (5×10^4^) were plated in the top chamber with Matrigel-coated membranes [Corning® BioCoat™ Matrigel® Invasion Chambers (Corning Costar, Cambridge, MA)]. Cells were resuspended in serum-free medium and added to the upper chambers. Medium containing 10% fetal bovine serum was added in the lower chamber. After incubation at 37 °C (24 h) the cells were fixed in chilled methanol for 30 min at 4 °C. Fixed cells were stained with 0.05% crystal violet solution for 30 min. The stained cells that invaded through the pores to the lower surface of the inserts were photographed and counted.

**Small interfering RNA (siRNA) transfection:** FOXO3 siRNA (sc-37887) and scrambled siRNA (sc-37007) were purchased from Santa Cruz Biotechnologies. Transfection of siRNAs was carried out using Lipofectamine 3000 reagent (Invitrogen, Carlsbad, CA, USA) as per the manufacturer's instructions. Briefly, SW-480-FUR or HCT-116 FUR cells (0.5 × 10^6^ cells/well) in 6 well plate were cultured overnight. These cells were transfected with FOXO3 siRNA or scrambled siRNA (200 pmol). The efficiency of transfection was confirmed by Western blot analysis. After 36 h of transfections, cells were treated with vehicle (0.01% DMSO), 5FU (50 µM), UroA (50 µM), UAS03 (50 µM), 5FU+UroA and 5FU+UAS03 for 24 h. The cell lysates were prepared, and levels of relevant proteins were assessed by Western blots. The apoptosis and cell proliferation of these cells were evaluated by Annexin V/PI and Ki-67 staining, respectively.

**Tumor xenograft model:** Xenograft tumors were generated by subcutaneous injection of 6 × 10^6^ HCT116-5FUR cells (in 100 μL of 1XPBS) into the flanks of 6-7 week-old NRGS mice. When palpable tumors formed, mice were randomly divided into 6 groups (n = 5-7) and treatment was initiated. Groups were as follows: (i) Vehicle (1% CMC, 0.1% Polysorbate 80) (ii) 5FU (20 mg/kg) (iii) UroA (40 mg/kg) (iv) UAS03 (40 mg/kg) (v) 5FU+UroA (vi) 5FU+UAS03. 5FU treatment was *i.p.*, whereas UroA and UAS03 were given orally three times a week. UroA was prepared in 1% CMC, 0.1% Polysorbate 80 and UAS03 was prepared in 1% CMC. A total of eight doses were administered. Tumor volume was measured every 2 days using calipers. The subcutaneous tumor volume (V) was calculated using the formula: V = L× W^2^ × 0.5, where L is length and W is the width of a tumor. Mice were euthanized when the vehicle group reached the endpoint criteria as per IACUC regulations. At the end of the study, all animals were sacrificed, and their primary tumors were excised from the body and the weight and size of each tumor was recorded.

**Histopathology and immunohistochemistry for tumor:** The tumors were fixed in 10% buffered formaldehyde solution overnight followed by 70% alcohol. Fixed tissues were processed for standard histopathological methods for paraffin embedding and 5 µm paraffin sections were cut and H and E stained by Horus Scientific (MA, USA). Brightfield images were captured using PanDesk Slide Scanner (3DHISTECH Ltd., MI, USA). The immunohistochemistry and immunofluorescence staining were performed using the methods described previously by our group 45, 46. Briefly, for immunofluorescence staining, the tumor sections were stained with primary anti-bodies (1:100 dilution) followed by Alexa flour 488 or Alexa flour 594 secondary antibody (1:500 dilution). Stained sections were mounted with VECTASHIELD HardSet™ antifade mounting medium with DAPI (Vector Laboratories) to stain the nuclei. The confocal images were captured using a Nikon A1R confocal microscope using appropriate laser channels. For staining of cleaved caspase 3 in tumor sections, the sections were stained with rabbit anti-cleaved caspase 3 antibody (1:100 dilution) using Super Sensitive™ Polymer-HRP detection System (BioGenex, CA, USA).

**AOM-DSS colon tumor model:** Seven-week-old C57BL/6 mice (n = 10/group; male and female) were used for the AOM-DSS colon tumor model. Briefly, C57BL/6 mice was divided into six groups as follows (i) Vehicle, (ii) 5FU (20 mg/kg) (iii) UroA (20 mg/kg) (iv) UAS03 (20 mg/kg) (v) 5FU+UroA (vi) 5FU+UAS03. Mice were injected with AOM (10 mg/kg; i.p.). After one week, mice were given 3 cycles of 1.5% DSS in drinking water (7 days 1.5% DSS followed 14 days recovery). Mice were weighed twice a week and were euthanized at day 70 following the AOM treatment. The treatment regimen was as follows: After the second DSS administration, 5FU (20 mg/kg) treatment (i.p.) was given twice a week for 5 weeks along with or without UroA or UAS03. Control groups of mice did not receive AOM or DSS. The percent body weight change for each mouse was evaluated with the following formula: [(W_x_ - W_0_)/W_0_] × 100%, where W_x_ is the mouse weight on day X, and W_0_ is the mouse weight at the start of treatment. The colons were separated, longitudinally opened and visible tumors were counted. The serum from these mice was used for measuring inflammatory cytokines (IL-6, TNF-α) using standard ELISA methods (Bio-legend).

**Statistical Analysis:** Statistics were performed using either unpaired t-test or ANOVA followed by Tukey's Multiple Comparisons Test using Graphpad Prism 9 (GraphPad Software, San Diego, USA) software. Details of the specific statistical tests are provided in the figure legends. Studies were performed in quadruplicates unless otherwise stated in the figure legends. Error bars, ±SEM; ****p < 0.0001; ***p < 0.001; **p < 0.01; *p < 0.05; ns: not significant.

## Results

### UroA and UAS03 sensitize 5FU resistant (FUR) colon cancer cells to 5FU treatment

**Figure 1:**
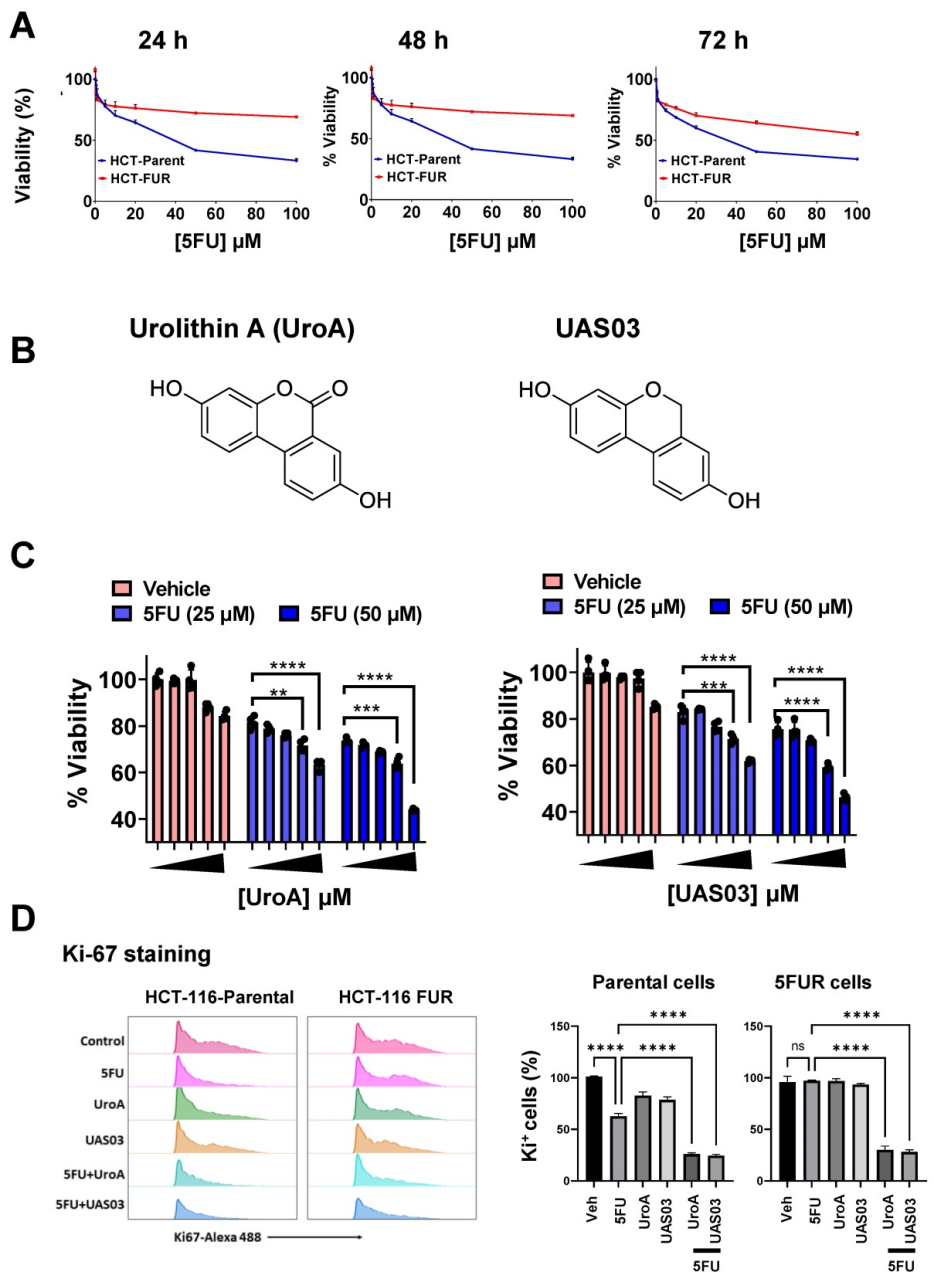
** UroA and UAS03 chemosensitize the 5FUR colon cancer cells. A.** HCT-116-parent and HCT-116-FUR cells (7,000 cells/well) were plated in 96 well plate and grown for overnight. Cells were treated with indicated compounds in a dose dependent manner for 24, 48 and 72 h. The cell viability was measured using standard MTT assay. **B.** Chemical structures of Urolithin A (UroA) and UAS03. **C.** HCT-116-FUR cell viability in co-treatments (UroA or UAS03 or 5FU at 50 μM or in combination) was calculated against DMSO (0.1%) as 100%.** D**. Combination therapy of 5FU with UroA/UAS03 effectively reduced cell proliferation**.** HCT-116-parental and HCT-116-FUR cells were treated with either 5FU (50 μM), UroA (50 μM), UAS03 (50 μM) or 5FU (50 μM) in combination of UroA (50 μM) or UAS03 (50 μM) for 24 h. Cells were stained with Ki67-Alexa488 and analyzed using flow cytometry. Flow cytometric analysis is represented by histograms and bar diagrams of Ki67^+^ cells. Data are represented as mean ± S.E.M. of three different experiments. Statistics performed one-way ANOVA using Graph Prism Software. ns: non-significant; ****P < 0.0001.

To investigate the effects of gut microbial metabolite, UroA and its analogue UAS03 on chemosensitization, we have utilized 5FU-resistant (FUR) and respective parental colon cancer cell line model systems. HCT116-FUR and SW480-FUR cell lines were generated by treating parental HCT116 and SW480 cell lines with 5FU for 9 months 47, 48. These cell lines display altered cellular morphology resembling their mesenchymal origin. As expected HCT-116 and SW480 FUR cells exhibited resistant to 5-FU treatment compared to respective parental cell line (Figure 1A and Figure S1A). We tested the hypothesis that microbial metabolite, UroA and UAS03 (Figure 1B) act as chemosensitizing adjuvants against 5FUR colon cancers. Dose range studies using UroA or UAS03 (0, 1, 10, 25, 50 μM) in combination with 5FU (25, 50 μM) suggested that UroA or UAS03 at 25 or 50 μM showed maximum anti-proliferative activities at 48 h (Figure 1C and Figure S1B). These results suggested that co-treatment with UroA or UAS03 chemosensitizes 5-FU resistant colon cancer cells to 5FU treatment. Anti-proliferative activities of 5FU in combination with UroA/UAS03 were also examined by measuring Ki-67 expression. As shown in Figure 1D, co-treatments (5FU+UroA or 5FU+UAS03) significantly reduced ki-67 positive cells in both HCT-116 parental and HCT-116-FUR cells suggesting decreased cell proliferation. Similar patterns were observed in SW480 cells (Figure S1C-D). To help elucidate the underlying mechanisms of UroA/UAS03 promoted anti-proliferative activity in combination with 5FU, we measured apoptosis after treating with either with UroA or UAS03 in combination with 5FU. Treatment with 5FU (50 μM) elicited apoptosis in the parental cell line, but not in 5FUR cells (Figure S2). A combination of UroA or UAS03 with 5FU significantly induced apoptosis in 5FUR cells (both in HCT-116 and SW480 cells) as evident from Annexin V/propidium iodide (PI)-staining. The representative flow cytometry data for HCT-116-FUR cells is shown (Figure S2A). The percentages of apoptosis upon treatments for both parental and 5FUR cells of SW480 and HCT-116 are represented in Figure S2B-C. Further, we evaluated expression of representative apoptosis markers (Bax, Caspase 9 and cleaved caspase 9) in these treatments. Bax and cleaved caspase 9 significantly increased in co-treatments (UroA/UAS03+5FU) compared monotreatment (Figure S2D). Overall, these studies suggest that UroA or UAS03 co-treatment with 5FU chemosensitizes and reduce cell viability, blocks cell proliferation and induce apoptosis of 5FUR colon cancer lines. Please note that the rationale for using UroA at micromolar concentration is that the plasma concentration of urolithins can reach micromolar levels without toxicity in humans 21, 25, 29, 49, 50. Therefore, micromolar doses of UroA were used in these *in vitro* experiments.

### UroA/UAS03 in combination with 5-FU reduce 5FUR invasion and regulate expression of epithelial-mesenchymal transition (EMT) mediators

**Figure 2:**
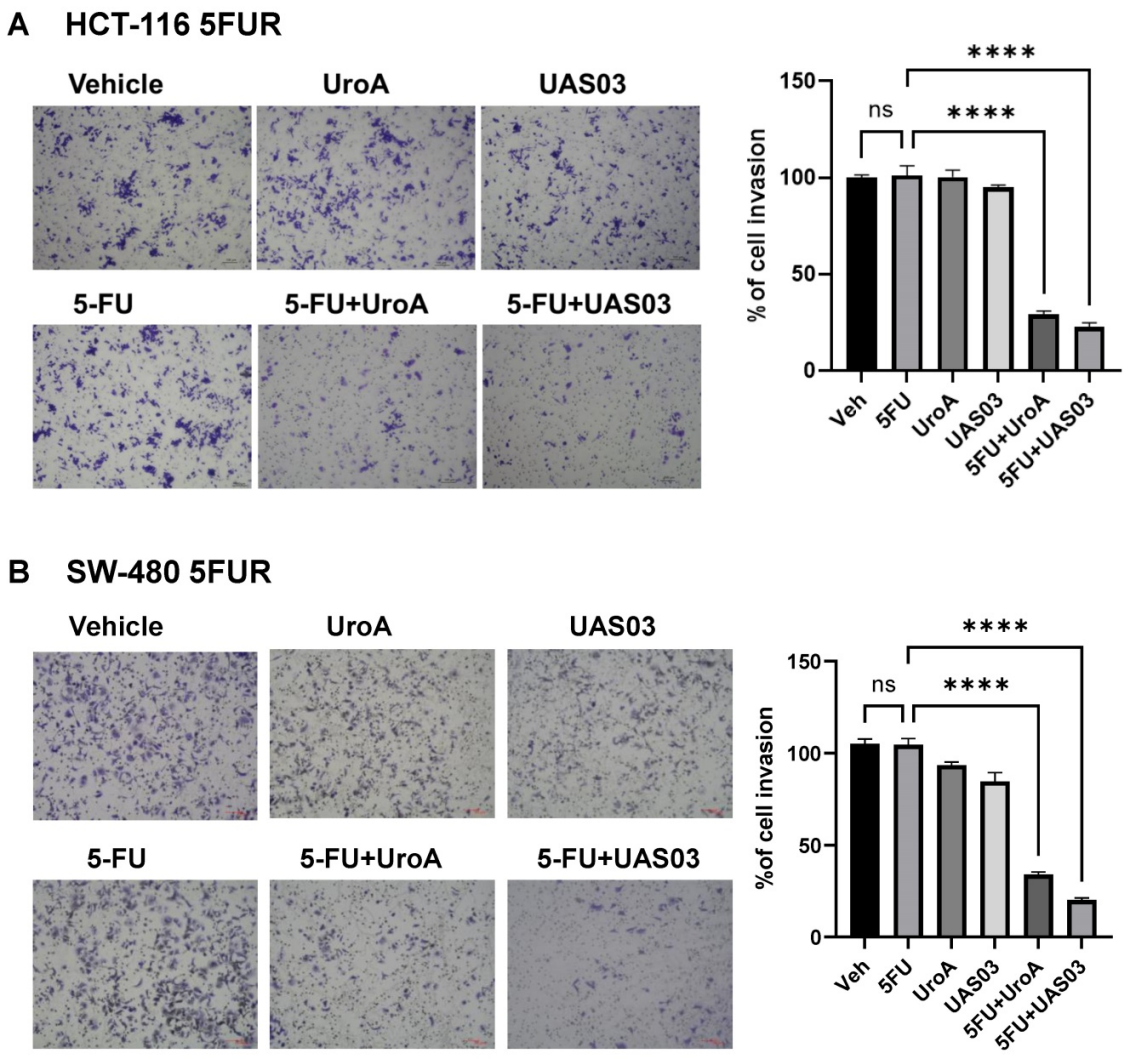
** 5FU in combination with UroA or UAS03 reduced invasion of 5FUR cells.** Invasion assay was carried out in 24-well transwell with HCT-116-FUR (A) or SW-480- FUR (B) cells treated with either 5FU (50 μM) or UroA (50 μM) or UAS03 (50 μM) or in combination for 24 h. The cells were stained with crystal violet. The randomly chosen fields were photographed (10X), and the number of cells migrated to the lower surface was calculated. Data are mean ± S.E.M. of four experiments. Statistics performed one-way ANOVA using Graph Prism Software. ns: non-significant; ****P < 0.001.

Since 5FUR cancers exhibit aggressive invasive properties, we tested whether UroA/UAS03 treatment regulates the cell migration and cell invasion in combination with 5FU. For this purpose, we have carried out standard scratch assay and 3D invasion experiments in HCT-116-FUR and SW480-FUR cells. Our results showed that UroA, UAS03 or 5 FU treatments alone did not impact either migration in scratch assay (Figure S3) or the invasion (Figure 2) of 5FUR cells. However, co-treatment 5FU+UroA or 5FU+UAS03 significantly reduced their migration and invasion abilities in both the cell lines (Figure S3, Figure 2). These results suggest that in addition to their anti-proliferative effects, co-treatments (5FU+UroA or 5-FU+UAS03) inhibited the migration of 5FUR colon cancer cells.

**Figure 3:**
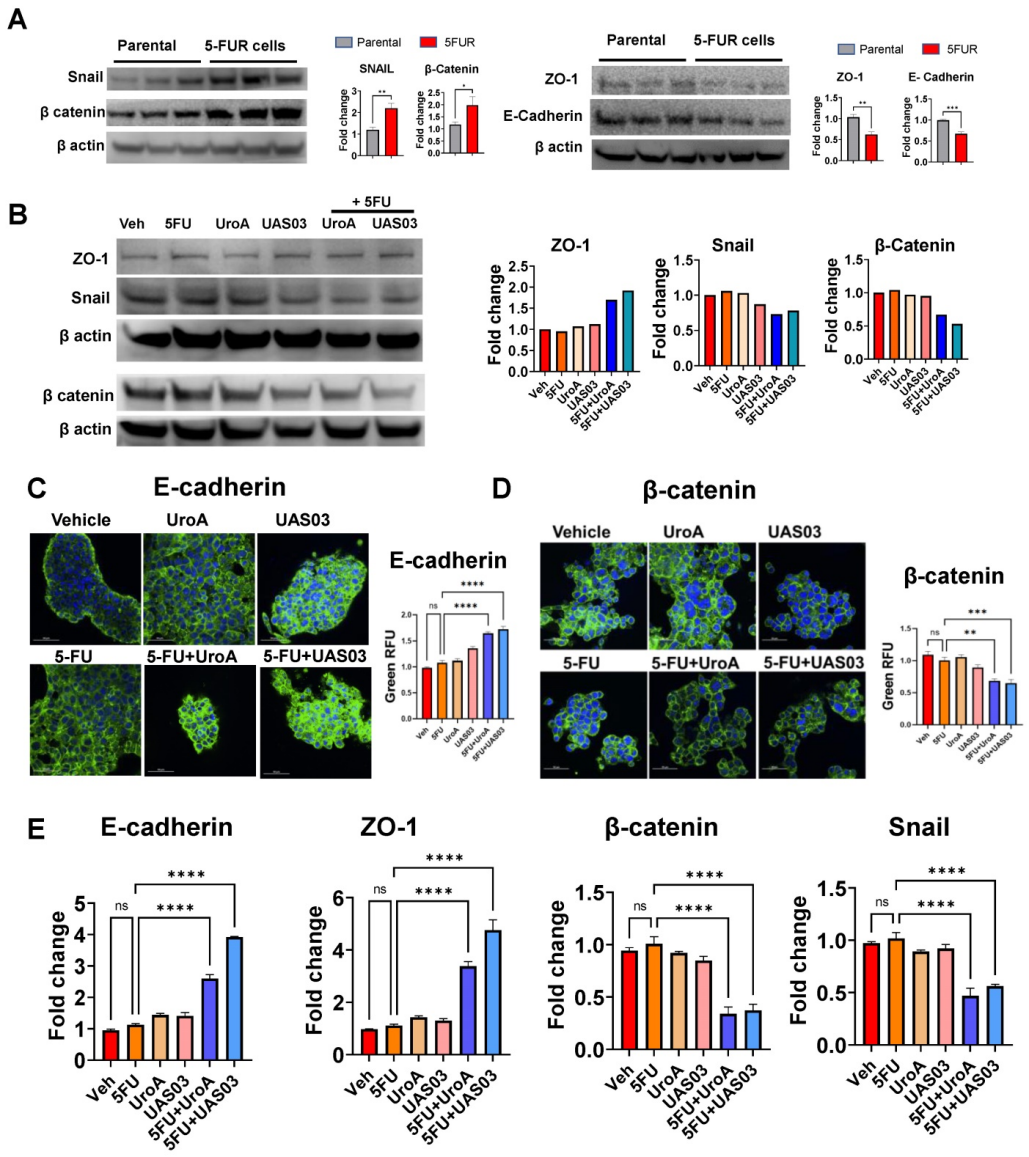
** 5FU modulates EMT markers upon co-treatment with UroA/UAS03. A.** Western blot analysis of EMT makers in HCT-116 parental and HCT-116-FUR cells. **B.** Western blot analysis of EMT makers in HCT-116-FUR cells treated with 5-FU (50 μM) in the presence of UroA (50 μM) or UAS03 (50 μM). **C-D.** HCT-116-FUR cells were grown on 8 well chamber slide and treated with either 5FU (50 μM), UroA (50 μM), UAS03 (50 μM) or 5FU (50 μM) in combination of UroA (50 μM) or UAS03 (50 μM) for 24 h. The cells were stained with either anti-E cadherin **(C)** or β catenin **(D)** followed by secondary antibody tagged with Alexa-488. Nucleus was stained using DAPI. The confocal images were captured. Scale bars indicate 50 μm. **E.** Total RNA was isolated from HCT-116-FUR cells and analyzed for the expression of E-cadherin, ZO-1, β-catenin and Snail. The fold changes in mRNA levels were determined by RT PCR method. Error bars, ±SEM. Statistics performed one-way ANOVA using Graph Prism Software. ns: non-significant, *p < 0.05, **p < 0.01, ***p < 0.001.

Previously, it was demonstrated that EMT, stemness, and the polycomb repressive complex are strongly correlated with increased migration and chemoresistance 51. Downregulation of E-cadherin is one of the hallmarks of EMT 52. To investigate the underlying mechanism by which UroA/UAS03 exert their anti-migratory activities, we examined the levels of several epithelial-mesenchymal transition (EMT) mediators following treatment of 5-FU-resistant cells with 5FU +/- UroA/UAS03. EMT markers such as β-catenin and Snail protein increased in SW480-FUR cells and HCT-116 FUR cells compared to their respective parental cells (Figure 3A, Figure S4A). In contrast, EMT protective proteins such as zona occludin 1 (ZO1) and E-cadherin are reduced compared to parental SW480 cells (Figure 3A, Figure S4A). Treatment with UroA or UAS03 in the presence of 5-FU led to an increase in the expression of ZO1 and E-cadherin compared to vehicle treatment (Figure 3B, Figure S4B). Conversely, UroA or UAS03 treatments decreased EMT markers such as β-catenin and Snail proteins in SW480-FUR cells. The expression levels of EMT mediators were confirmed by confocal imaging (Figure 3C-D) as well as at the mRNA level (Figure 3E, Figure S4C). Overall, these results suggest that UroA/UAS03 in combination with 5FU modulates the EMT process and reduces the invasion ability of 5FUR cancer cells.

### UroA and UAS03 treatment downregulate drug transporters

One of the mechanisms by which cancer cells acquire chemoresistance is by enhanced expression of efflux pumps such as ATP-binding cassette (ABC) drug efflux transporters, Breast Cancer Resistance Protein (BCRP), p-glycoprotein (p-gp), Multidrug resistance protein 2 (MRP2) and MRP7 53, 54. Blocking the activity and/or downregulating the expression of efflux pumps could potentially sensitize both drug-sensitive and drug-resistant cancer cells to anti-cancer treatments 55, 56. To test whether UroA/UAS03 regulate the levels of efflux pumps, we examined the expression levels and efflux pump activities in HCT-116 parent, HCT-116-5FUR, SW480 parent and SW480-FUR cells. Indeed, we found that HCT-116-5FUR and SW480-FUR cells display increased expression of several well-studied drug transporters (MDR, BCRP, MRP2) compared to parental cells at mRNA and protein levels (Figure S5, S6 A-B). Consistent with these data, the physiological activities of these efflux pumps are also significantly higher in FUR cells than parental cells (Figure S5C, S6C). Next, we examined the expression of drug transporters in these cells that were treated with UroA/UAS03 in the presence or absence of 5FU. Our results suggest that co-treatments significantly reduced expression of MDR and BCRP (Figure S7).

**Figure 4:**
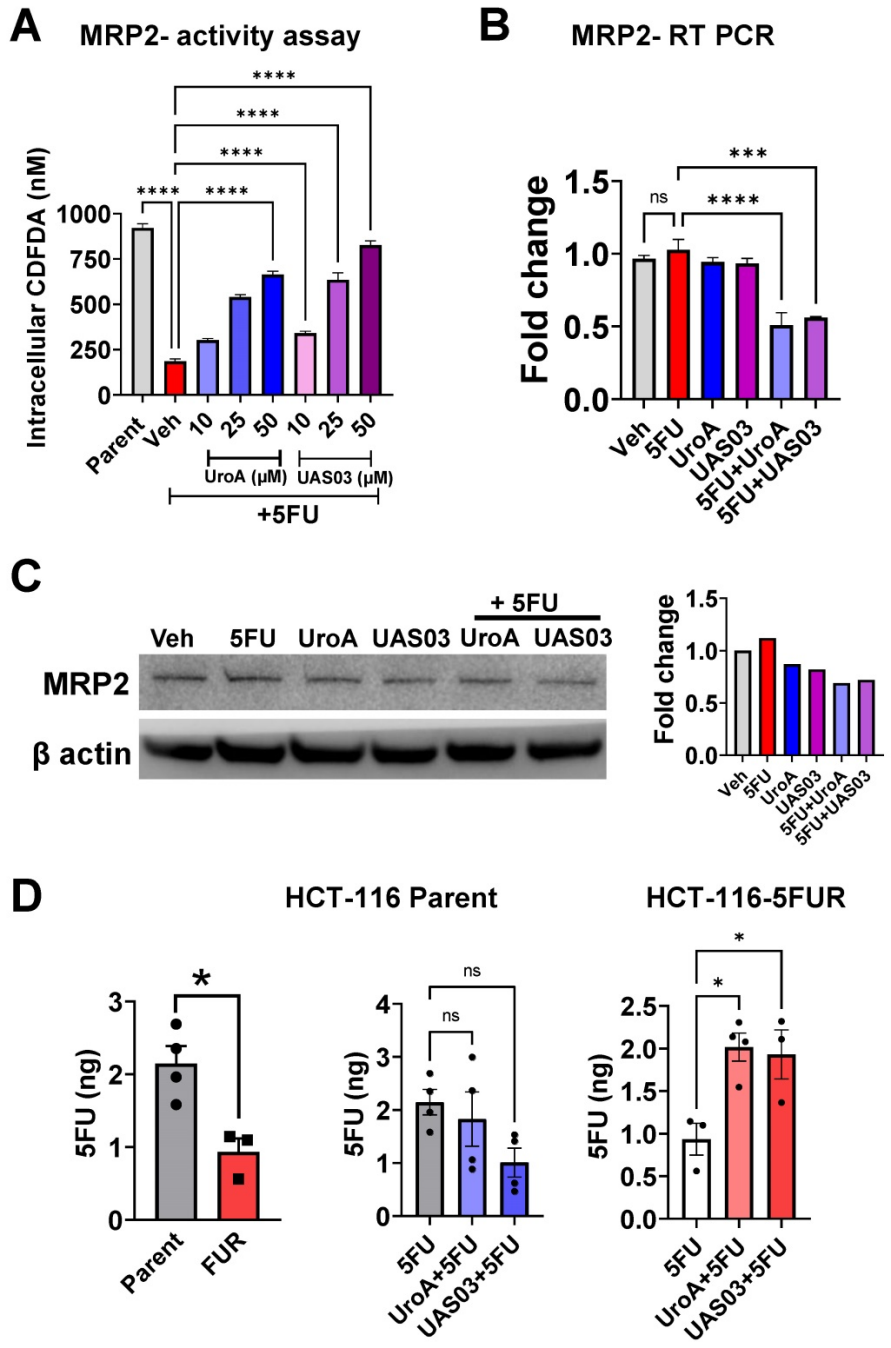
** Treatment with UroA and UAS03 reduce expression and activity of MRP2. A.** Parental HCT-116 and HCT-116 FUR cells were used to evaluate MRP2 efflux of CDFDA. MRP2 transporter assay was performed using CDCFDA substrate and measured intracellular CDCFDA after incubation 1 h in the presence of Vehicle (0.1% DMSO) or UroA or UAS03 and 5FU (50 μM)** B.** Parental HCT-116 and HCT-116 FUR cells were treated with vehicle (0.1% DMSO) or UroA or UAS03 (50 µM) and 5FU (50 μM) for 24 h. Total RNA was isolated and mRNA levels of MRP2 were determined by real time PCR. **C.** HCT-116 -FUR cells were treated with UroA or UAS03 in the presence of 5 FU (50 µM) for 24 h. MRP2 protein levels were measured by Western blots and quantified fold change. **D.** Levels of 5FU measured in HCT-116 parental and HCT-116 5FUR cells. Cells in 96 well plate treated with vehicle (0.1% DMSO) or UroA or UAS03 (50 μM) for 24 h and followed by 90 min of 5FU (50 μM) treatment. Total 5FU extracted in to 10% methanol and levels of 5FU determined using HPLC as described in methods. Error bars indicate ±SEM. Statistics performed using unpaired t-test. ns: non-significant. *p < 0.05, **p < 0.01, ***p < 0.001.

Since, 5FU is a well-known substrate for MRP2-mediated transport 57, we further examined the expression and functional activity of MRP2 in these colon cancer cell lines. HCT-116 parent or HCT-116-5FUR cells were treated with vehicle/UroA/UAS03 in the presence or absence of 5 FU at indicated doses for 24 h. As shown in Figure 4, co-treatment with UroA/UAS03 and 5FU decreased MRP2 efflux activity as evident from increased levels of dose-dependent intracellular CDCFDA (MRP2 transporter assay) (Figure 4A)**.** UroA or UAS03 also significantly reduced MRP2 at the mRNA and protein levels (Figure 4B and 4C). The similar results were observed in SW480 parent or SW480-FUR cells (Figure S8). These studies showed that UroA and UAS03 treatment sensitized the 5FU resistant cells by downregulating drug transporters.

Next, we measured intracellular 5FU levels to directly determine the effects of UroA or UAS03 on efflux of 5FU in HCT-116 parental and HCT-116 FUR cells as representative cell line. As shown in Figure 4D that intracellular levels of 5FU are significantly higher in parental cells than 5FUR HCT-116 colon cancer cells. Treatment with UroA or UAS03 led to a significant increase in 5FU levels in HCT-116 5FUR cells suggesting co-treatment lowered the efflux of the 5FU. Increased levels of 5FU potentially can be attributed to decreased expression of drug transporters upon treating with UroA or UAS03.

### UroA and UAS03 treatment regulate drug transporters through the FOXO3-FOXM1 axis

**Figure 5:**
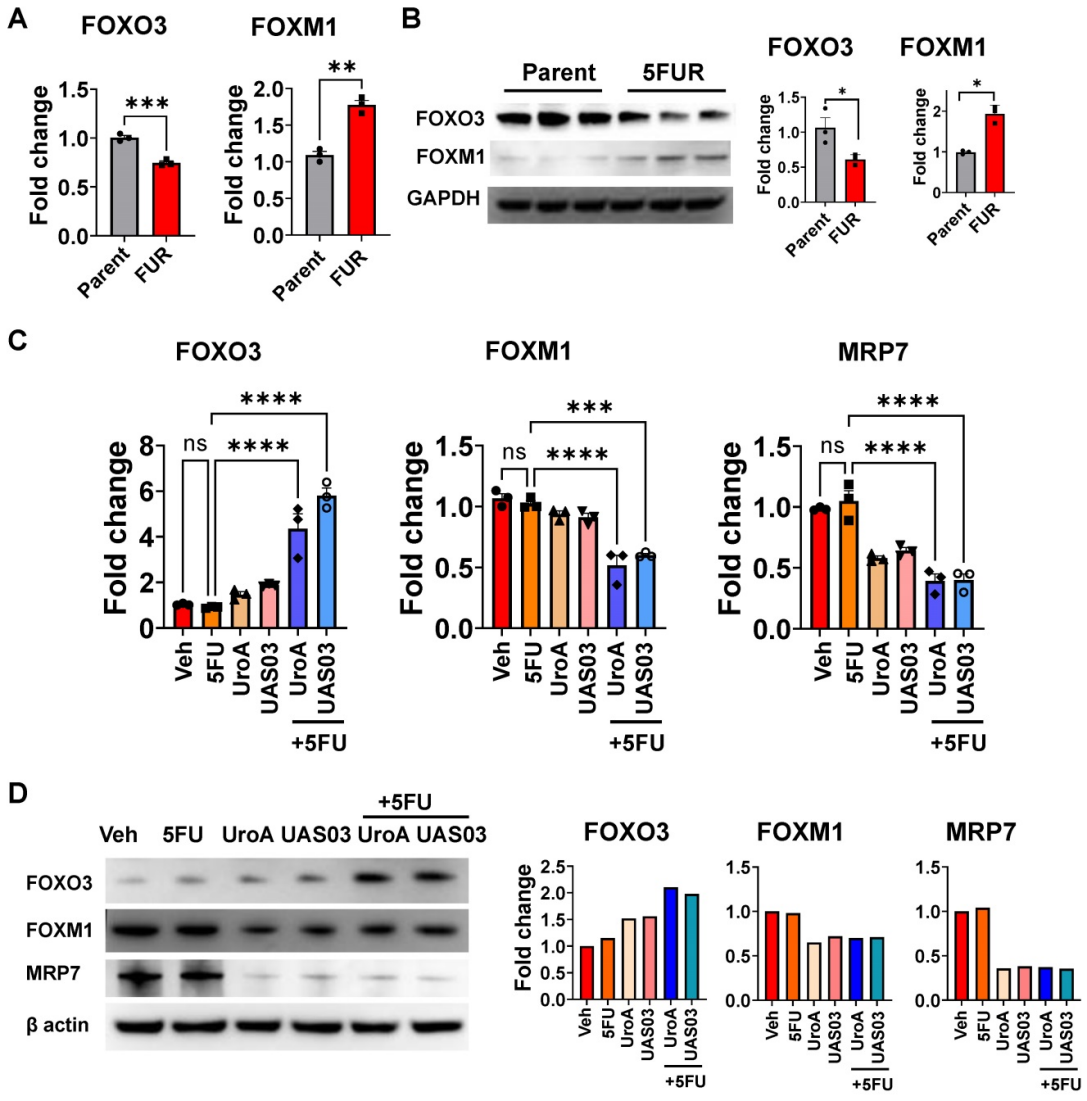
** Treatment with UroA/UAS03 regulates the FOXO3-FOXM1 axis. A.** Total RNA was isolated from parental HCT-116 and HCT-116-FUR cells and analyzed expression of FOXO3 and FOXM1.The fold changes in mRNA levels were determined by RT PCR method. **B.** Western blot analysis of FOXO3 and FOXM1in parental HCT-116 and HCT-116-FUR cells. **C.** Total RNA was isolated from HCT-116-FUR cells treated with either 5FU (50 μM) or UroA (50 μM) or UAS03 (50 μM) or in combination for 24 h and analyzed for the expression of FOXO3, FOXM1 and MRP7 by SyBR RT PCR. The fold changes in mRNA levels were represented. **D.** HCT-116-FUR cells were treated with either 5FU (50 μM) or UroA (50 μM) or UAS03 (50 μM) or in combination for 24 h. Western blot analysis of FOXO3, FOXM1 and MRP7. Statistics performed one-way ANOVA using Graph Prism Software. Error bars, ±SEM. **p < 0.01, ***p < 0.001.

The forkhead box O3 (FOXO3) and forkhead box M1 (FOXM1) are transcription factors, which are known upstream regulators of drug transporters 58. FOXO3 and FOXM1 play crucial role in physiological functions including cell proliferation, differentiation, cell survival, senescence, DNA damage repair and cell cycle control 58. Increased overexpression of FOXM1 and decreased expression of FOXO3 are associated with resistance to cancer therapeutics 59. FOXO3 inactivates FOXM1 directly and antagonizes FOXM1 function by competing for the same target genes 60. Previous studies reported that in case of 5FU resistant in colon cancer, both FOXO3 and FOXM1 play critical roles 61, 62. Therefore, we hypothesized that UroA/UAS03 co-treatment with 5FU regulates the FOXO3-FOXM1 axis leading to downregulation of drug transporters. As shown in Figure 5A-B, HCT-116 5FUR cells display downregulation of FOXO3 and increased expression of FOXM1 compared to parental colon cancer cell lines. Treatment with UroA or UAS03 alone significantly upregulated FOXO3 and down regulated FOXM1 in 5FUR cells both at the mRNA and protein levels (Figure 5C and 5D). Further, UroA/UAS03 co-treatment with 5FU significantly upregulated expression of FOXO3 and downregulated FOXM1 both at mRNA and protein levels. The cross-regulation of FOXO3 and FOXM1 is more prominent in co-treated samples. FOXM1 is known to regulate drug transporter ABCC10 (MRP7), which is critical for acquiring 5FU resistance 62. Analysis of MRP7 expression suggested that UroA or UAS03 treatment significantly downregulated MRP7 in 5FUR colon cancer cells (Figure 5C-D). The similar results were observed in SW480-5FUR cells (Figure S9). Thus, we conclude that it is possible that UroA/UAS03 chemosensitizes 5FUR cells by downregulating the expression of drug transporters via modulation of the FOXO3-FOXM1 transcription factors.

**Figure 6:**
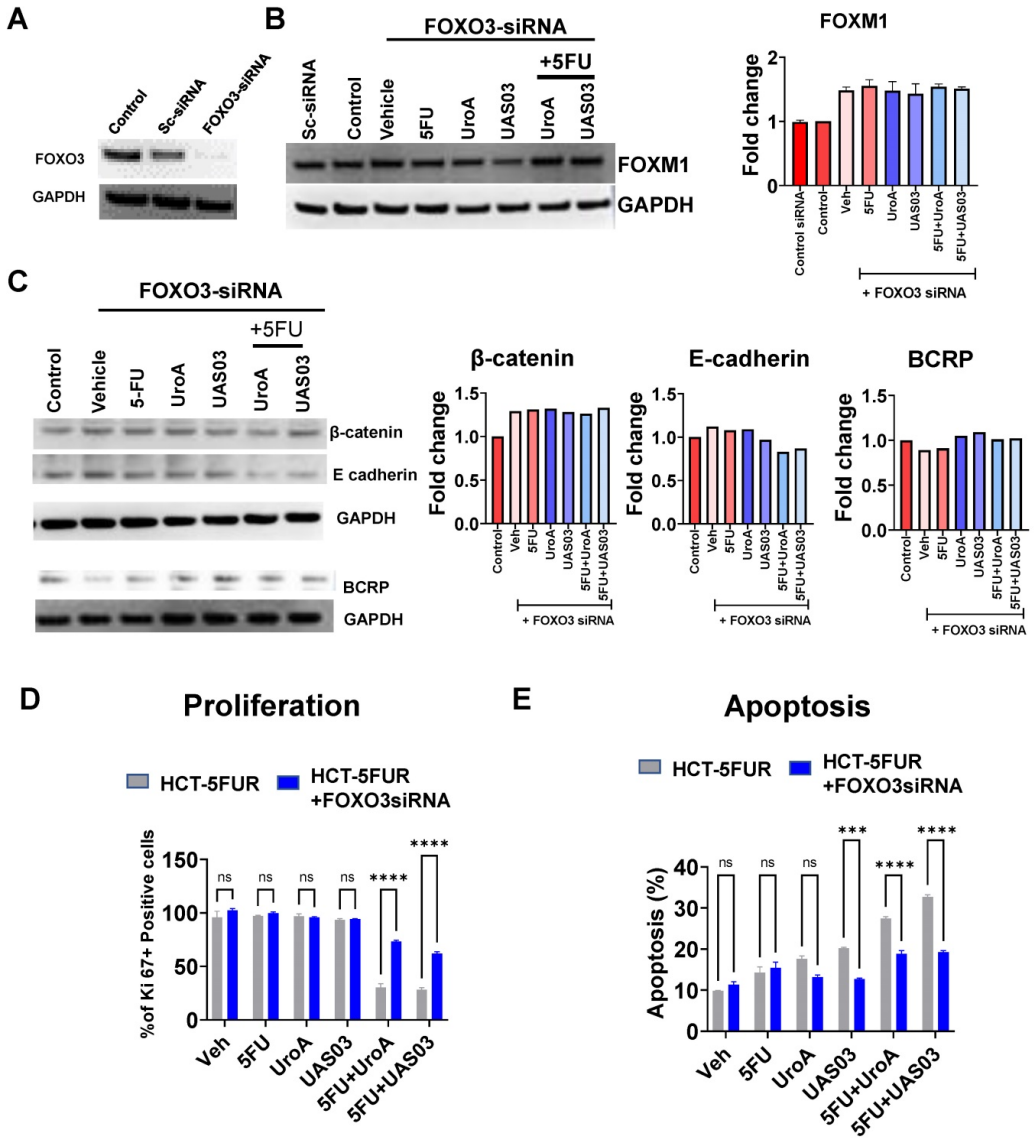
**Knockdown of FOXO3 abrogated UroA/UAS03-mediated chemosensitization activities. A**. FOXO3 was knockdown in HCT-116-5FUR cells using FOXO3-siRNA. Expression of FOXO3 was confirmed by Western blot. **B-E.** These cells were treated with either 5FU (50 μM) or UroA (50 μM) or UAS03 (50 μM) or in combination for 24 h and expressions of FOXM1 (**B),** β-catenin, E-cadherin and BCRP **(C)** were determined by Western blots. Cell proliferation by Ki67 staining **(D)** and apoptosis by Annexin V and PI staining using flow cytometry methods (**E).** Statistics were performed one way ANOVA using Graph Prism Software. Error bars, ±SEM. **p < 0.01, ***p < 0.001.

To obtain direct evidence that FOXO3 plays a key role in UroA/UAS03-mediated chemosensitization mechanisms, the expression of FOXO3 was knocked down in HCT 116-FUR or SW480 5FUR cells using FOXO3 specific siRNA (Figure 6, Figure S10). Indeed, FOXO3 knockdown led to an increase in expression of FOXM1, consistent with the observation that FOXO3 is a negative regulator of FOXM1 (Figure 6B, Figure S10A). Treatment with UroA or UAS03 or in combination with 5FU did not alter the expression of EMT markers (β-catenin, E-cadherin) or drug transporters (BCRP) in FOXO3 knockdown cells compared to scrambled siRNA transfected cells (Figure 6C). Knockdown of FOXO3 resulted in slightly decreased expression of E-cadherin in co-treatments (5FU+UroA/UAS03), which suggests the requirement of FOXO3 for UroA/UAS03 mediated upregulation of E-cadherin in combination with 5FU. The functional consequences of knockdown of FOXO3 were assayed by measuring proliferation and apoptosis. Our results showed that UroA/UAS03 in combination with 5FU failed to reduce cell proliferation (Figure 6D) or induce apoptosis (Figure 6E) in FOXO3 knockdown cells. The similar results were observed in SW480-5FUR cells (Figure S10B and S10C). In summary, these studies indicate an important role for FOXO3 in UroA/UAS03-mediated regulation of expression of drug transporters and EMT markers, as well as chemosensitization mechanisms.

### UroA or UAS03 treatment sensitizes 5FUR tumors to 5FU treatment in mice

**Figure 7:**
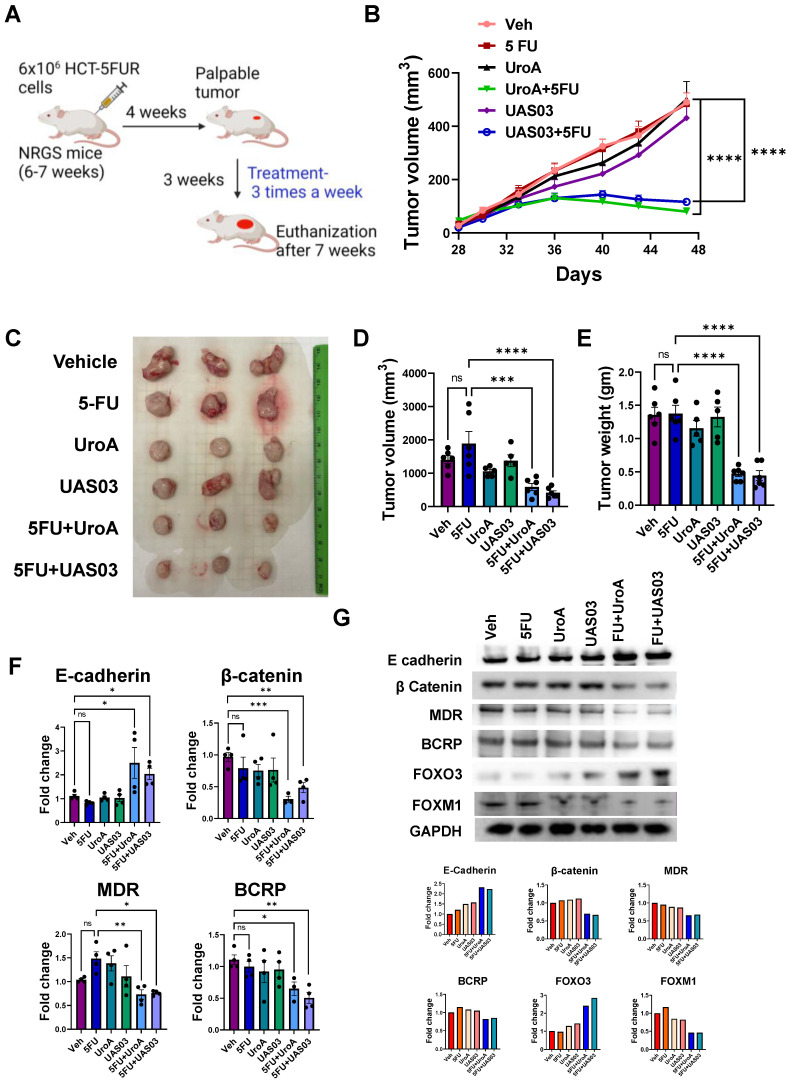
** UroA/UAS03 co-treatment with 5FU reduce 5FUR tumors in mice. A.** Schematic representation of treatment protocol in HCT-116-FUR tumor bearing NRGS mice NRGS mice (n=5-7 per group) were s.c. implanted with HCT-116-FUR and subjected to indicated treatments. **B.** Change in tumor volume during treatment period**. C.** Representative gross images of xenograft tumor **D-E.** Xenograft tumors from dissected mice were measured for tumor volume and tumor weight.** F.** Total RNA was isolated from tumors and E-cadherin, β Catenin, MDR and BCRP expressions were analyzed. The fold changes in mRNA levels were determined by RT PCR method. **G.** Protein level expression of E-cadherin, β Catenin, MDR, BCRP, FOXO3 and FOXM1 in the tumors were measured by immunoblots and quantified. Error bars, ±SEM; Statistics performed one way ANOVA using Graph Prism Software. ns: non-significant, ****p < 0.0001; ***p < 0.001; **p < 0.01 *p < 0.05.

To determine whether UroA and UAS03 exhibit chemosensitizing effects *in vivo*, immunodeficient NRGS mice were implanted with HCT-116-5FUR cells (s.c) to generate solid 5FU-resistant tumors. Since UroA/UAS03 in combination with 5FU showed similar impact on HCT-116 and SW480 cells (*in vitro*), we selected HCT-116-5FUR cell line to generate implantable tumors in NRGS mice to examine the impact of combination therapy. NRGS mice (also known as NRG-SGM3, NRG-3S) mice are NOD.*Rag1^-/-^;γc^null^* (NRG) animals expressing human interleukin-3, human granulocyte/macrophage-stimulating factor, and human Steel factor from the SGM3 (3GS) triple co-injected transgenes NRGS mice 63. Palpable tumors appeared around 4 weeks post implantation. Mice were treated with 5FU or DMSO (0.05%) in combination with vehicle (1% CMC, 0.1% Tween-80) or with UroA or UAS03. The mice were treated 3 times a week until the end point of the experiment when tumors reached 500 mm^3^ in the vehicle control group (~7 weeks post-implantation) (Figure 7A**)**. As shown in Figure 7B, treatment with 5-FU alone did not decrease the tumor burden/size and looked similar to the vehicle treatment, suggesting that these tumors are indeed 5FU resistant. As expected, treatment with either UroA or UAS03 alone (40 mg/kg by oral gavage) did not reduce the tumor burden in these mice. However, treating the mice with 5FU in combination with UroA or UAS03 significantly reduced tumor size in these mice (Figure 7B). It is more evident from surgically removed tumors (Figure 7C) that tumor volume (Figure 7D) and weight (Figure 7E) are significantly reduced by co-treatment with 5FU + UroA/UAS03. Furthermore, co-treatment had a significant effect on the levels of EMT markers, where E-cadherin was upregulated and β-catenin downregulated in the tumors, thereby corroborating the *in vitro* studies described above (Figure 7F-G, Figure S11). Next, we analyzed the expression of drug transporters in these tumors. As expected, co-treatment (5FU+UroA or 5FU+UAS03) significantly reduced the expression of several drug transporters (MDR, BCRP), both at the mRNA (Figure 7F) and protein levels (Figure 7G). In corroboration with *in vitro* studies, co-treatment (5FU + UroA/UAS03) led to increase in the expression of FOXO3 and reduced the expression of FOXM1 in 5FUR xenograft tumors compared to mono treatments (Figure 7G). Additionally, we characterized the apoptosis marker, cleaved caspase 3 on tumors from these mice. As shown in Figure S12, co-treatments (UroA/UAS03 + 5FU) significantly increased apoptotic marker (Figure S12A) as well as necrotic regions (Figure S12B) in the tumors. These studies indicate that down regulation of drug transporters during co-treatment (UroA/UAS03 + 5FU) enable effective elimination of 5FUR cancer cells potentially through FOXO3-FOXM1 signaling leading to reduced tumor size.

### Combination therapy mitigates AOM-DSS induced colon tumors

**Figure 8:**
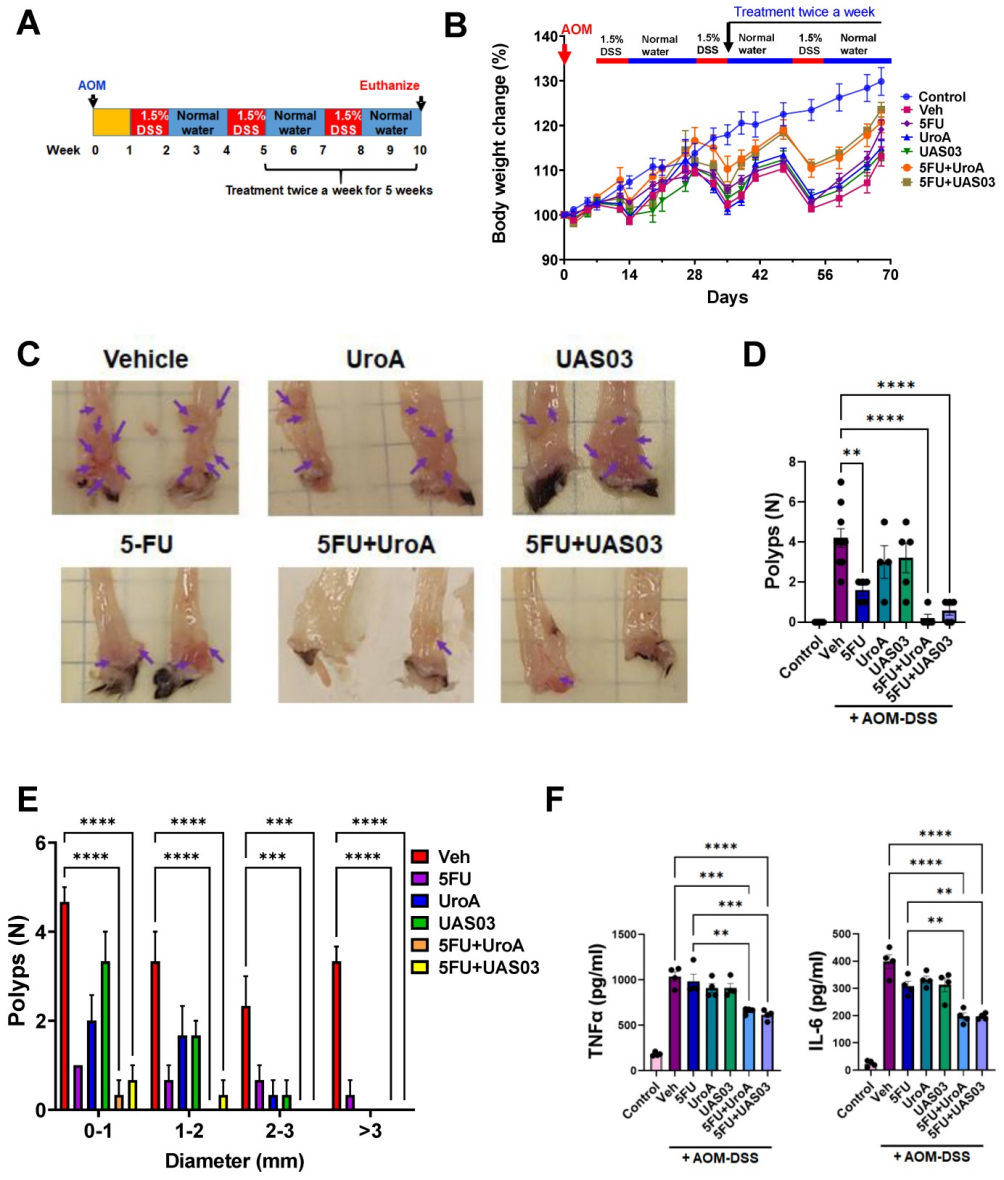
** Combination therapy mitigates AOM-DSS induced colon tumors. A.** Schematic representation of AOM-DSS model with treatment. Seven-week age-old C57BL/6 mice (n=10 per group) were subjected to AOM-DSS induced colon tumor model and subjected to indicated treatments.** B.** Body weight change graph **C.** Representative gross images of distal colons from mice treated with AOM-DSS. **D.** Total number of polyps was counted in colon of mice.** E.** The size of polyps in colon were determined and the average frequency of polyp distribution is shown. **F.** SerumTNF-α and IL-6 levels were estimated by ELISA. Error bars, ±SEM; Statistics performed one way ANOVA using Graph Prism Software. ****p < 0.0001, ***p < 0.001; **p < 0.01.

Finally, we tested whether the anti-tumor activity of combination therapy (5FU+UroA/UAS03) is effective in the well-studied AOM-DSS induced colon tumor model (15). Mice were injected i.p. with a single dose of AOM (10 mg/kg) followed by 3 cycles of 1.5% DSS in the drinking water alternating with 14-days of regular water (Figure 8A). The treatment started at week 5 post-AOM (twice a week) until 10 weeks when the experiment was terminated. Similar to the xenograft treatments described above in Figure 7, mice were divided into six groups and treated with vehicle or 5FU or UroA or UAS03 or 5FU+UroA or 5FU+UAS03. 5FU (20 mg/kg) treatment (i.p.) was given twice a week for rest of the 6 weeks along with or without UroA or UAS03 (oral gavage). Figure 8B shows that combination therapy protected mice from bodyweight loss compared to vehicle or single treatments (5FU or UroA or UAS03). Analysis of colon tumors in these mice suggested that treatment with 5FU reduced tumor burden. Interestingly, however, treatment with UroA or UAS03 also reduced the polyp number. Importantly, 5FU treatment combined with either UroA or UAS03 dramatically reduced tumor numbers and size (Figure 8C-E). In some cases, combination therapy completely abrogated colon tumors in these mice. The polyps that were observed following combination therapy were very small and ranged from 0-1 mm suggesting effective elimination/blocking of the progression of tumors (Figure 8E). Furthermore, analysis of inflammatory mediators in the serum of these mice suggested that co-treatment significantly reduced TNF-α and IL-6 compared to vehicle or single treatments (Figure 8F). Mice subjected to the AOM-DSS model develop splenomegaly and enlarged mesenteric lymph nodes due to increased inflammation. In agreement with previous observations, mice subjected to AOM-DSS and treated with vehicle displayed splenomegaly and swollen mesenteric lymph nodes (Figure S13). Consistent with tumor development, mice treated with 5FU or UroA or UAS03 alone exhibited splenomegaly and swollen lymph nodes (Figure S13). Co-treatment (5FU+ UroA or 5FU+UAS03) protected the mice from developing splenomegaly or swollen lymph nodes, suggesting that combination therapy regulates overall inflammation in this model apart from blocking tumor development (Figure S13).

## Discussion

The standard therapy for the past 60 years to treat CRC is surgery plus chemotherapy involving 5FU treatment. Despite many advantages of 5FU, the overall response rate for advanced CRC is only 10-15%. However, in combination with other anti-tumor drugs, the response rate improved to 40-50%. One of the major challenges of long-term chemotherapy with 5FU is the development of 5FU resistant (5FUR) cancers. Therefore, new therapeutic strategies are urgently needed to treat CRC. Growing evidence suggests that microbial dysbiosis is related to poor survival and prognosis of colon cancer patients 64. Moreover, it has been shown that the presence of certain microbiota is critical for effective chemotherapeutic outcomes 65-67. Here, we tested whether microbial metabolites such as UroA and its structural analogue (UAS03) can overcome 5FU chemoresistance and regulate cancer progression.

Our results suggest that UroA or UAS03 synergistically act with 5FU to increase colon cancer cell death in both 5FU-sensitive and 5FU-resistant cancer cell lines. Specifically, UroA or UAS03 co-treatment with 5FU significantly enhanced the chemotherapeutic efficacy of 5FU in 5FUR colon cancer cells as evident from decreased cell survival, increased apoptosis, as well as cell migration. Hallmarks of aggressive cancers include increased cell migration and EMT. The data presented here show that co-treatment with UroA or UAS03 plus 5FU attenuated cell migration both in 2D and 3D culture as well as modulated the EMT signature for better prognosis. Potential mechanisms for 5FU chemoresistance include alteration of drug influx and efflux, enhancement of drug inactivation and mutation of the drug target. In addition, long-term treatment with 5FU leads to increased expression of thymidylate synthase (TS), Bcl-1, Bcl-XL and Mcl-1 as well as increased activity of deoxyuridine triphosphatase and methylation of MLH1, which are associated with 5FU resistant colon cancers (reviewed in 68). Among these possibilities, increased expression of drug transporters (MDR, ATP-binding cassette (ABC) transporters) has been considered to be one of the prominent mechanisms responsible for 5FUR colon cancers 69. In this study, we showed that UroA/UAS03 mediates regulation of drug transporter proteins that presumptively to sensitizes colon cancer cells to 5FU treatment.

Drug transporters assist in the translocation of various molecules/substrates such as ions, sugars and amino acids across cellular membranes using ATPases and channel proteins. Previous studies reported that the expression of drug transporter encoding genes is significantly upregulated in chemoresistance colon cancers 70. In 5FUR cancers, increased expression of drug transporter proteins reduces the intracellular 5FU concentration by increasing 5FU efflux. In agreement with previous reports, our analysis showed that expression of drug transporters is significantly increased in 5FUR cancer cells compared to parental cell lines. Conversely, combination therapy (5FU+UroA/UAS03) significantly downregulated these drug transporter proteins' expression and functional activities. Consistent with the conclusion that drug transporters play a key role in 5FU resistance, intracellular 5FU is significantly higher in parental (5FU sensitive) colon cancer cells compared to 5FUR cells and pre-treatment with UroA or UAS03 significantly increased intracellular 5FU concentrations in 5FUR cells. In these experiments, 5FU measurements were made at a single time point (90 min post 5FU treatment) to avoid the complication of the potential metabolism of 5FU. Irrespective of this relatively short incubation time, our methodology successfully identified 5FU in cell lysates. Our data demonstrated that the treatment with UroA/UAS03 increased intracellular 5FU concentrations in 5FUR cells compared to vehicle. Interestingly, UroA/UAS03 treatment did not result in the increase of intracellular 5FU in parental HCT-116 cells. Despite differences between vehicle and UroA/UAS03 are not significant, the trend seems to be decreasing the overall intracellular 5FU in parental cells. We predict that UroA/UAS03 co-treatment in combination with 5FU may potentially lead to homeostatic levels of drug transporters such that 5FU is released out quickly or increased apoptosis may have led to dilution of 5FU. Further studies are warranted such as time and dose dependent treatments to understand the mechanisms of this process.

FOXO3 and FOXM1 are forkhead box transcription factors known to regulate several genes involved in cell cycle regulation, apoptosis, metastasis, DNA damage repair, senescence and drug resistance 58, 71. These transcription factors function downstream of the PI3K-Akt, Ras-ERK and JNK/p38MAPK signaling cascades, which are involved in important cellular processes that govern cell fate 71, 72. Previous studies showed that the FOXO3-FOXM1 axis plays a critical role in developing chemoresistance 58, 73, 74. FOXO3 is negatively regulated by activation of PI3K-Akt, Ras-ERK and ERK/MAPK 75. Because FOXO3 assists in eliminating damaged or early transformed cells through cell death and senescence; FOXO3 is considered a *bona fide* tumor suppressor protein 76. Our studies suggest that FOXO3 expression is significantly downregulated in 5FUR colon cancer cells compared to parental colon cancer cells. Combination therapy (5FU+UroA or 5FU+UAS03) led to an increase in the expression of FOXO3 both in cell lines and 5FUR tumors. It is known that activated Akt and ERK kinases (upregulated in 5FUR cells) negatively regulate FOXO3 by inducing nuclear exclusion of FOXO3 to the cytoplasm, where FOXO3 undergoes degradation 77. Conversely, it has been shown that the expression of functional FOXO3 enhanced the chemosensitivity of colon cancer cells to cisplatin treatment 77. Importantly, the PI3K/mTOR inhibitor NVP-BEZ235 effectively reversed 5-FU resistance through inactivation of PI3K/Akt, which led to enhanced levels of FOXO3 in colon cancer cells 78. Previously, we have shown in collaboration with Dr. Nagathihalli's group that treatment with UroA suppresses the PI3K/AKT/mTOR pathway in pancreatic ductal adenocarcinoma (PDAC) 79. Consistent with these results, it was shown that UroA treatment of PDAC cells blocked the phosphorylation of AKT and p70S6K *in vitro,* successfully inhibiting the growth of tumor xenografts, and increased overall survival of Ptf1a^Cre/+^;LSL-Kras^G12D/+^;Tgfbr2^flox/flox^ (PKT) mice compared with vehicle or gemcitabine therapy 79. It is possible that treatment with UroA/UAS03 reduces Akt or ERK directly activates FOXO3 through a Nrf2-dependent pathway in 5FUR cells.

We have mined the expression patterns of FOXM1, FOXO3, MRP2 and MRP7 genes in tissues of human colon cancers and normal tissues using Gene Expression database of Normal and Tumor tissues 2 (GENT2) (http://gent2.appex.kr) data base 80. In this resource authors utilized two platforms U133Plus2 (GPL570) and U133A (GPL96). The GPL570 contains 3775 colon cancer tissues and 397 normal colon cancer tissue samples, whereas the GPL96 data set contains 1112 colon cancer and 127 normal colon cancer tissues. As shown in Figure S14 that FOXM1, MRP2 and MRP7 significantly increased colon cancer tissues compared to normal tissues. FOXO3 significantly decreased in colon cancer tissues compared to normal colon tissues. It is pertinent to present data from 'GEPIA: a web server for cancer and normal gene expression profiling and interactive analyses' 81 that increased expression of MRP2 and MRP7 are associated with decreased survival of colon cancer patients. Please note that this data was not stratified as 5FU sensitive vs 5FU resistance cancer types due to unavailability of information from these data sets. Capturing information about drug resistance vs sensitive cancers and direct comparison of FOXO3-FOXM1 axis with drug transporters would provide better understanding of chemoresistance mechanisms in colon cancer. Further, the clinical relevance of our findings needs to be established.

Previously, we have shown that treatment with UroA/UAS03 enhances gut barrier function through AhR-Nrf2-dependent pathways 39. AhR modulates Nrf2-activation and transcriptional regulation of anti-oxidant proteins such as NAD(P)H:quinone oxidoreductase-1 (NQO1) (reviewed in 82). Moreover, it has been shown that FOXO3 modulates negative regulator of Nrf2, Kelch-like ECH-associated protein 1 (Keap 1) 83. The role of Nrf2 in cancer is debatable. It may function in a pro-oncogenic or anti-oncogenic manner depending on a variety of factors 84. In a study of melanoma cells, curcumin-induced oxidative stress led to the phosphorylation of FOXO3 by Mst1, which triggers FOXO3 and 14-3-3 dissociation resulting in nuclear translocation of FOXO3, thus initiating the transcription of FOXO3 target genes 85. Based on our observations and the literature, we postulate that UroA/UAS03-mediated Nrf2 activation leads to induction of macrophage stimulating 1 (Mst1) leading to FOXO3 activation. FOXM1 is a potent oncogene and one of the most crucial downstream targets of FOXO3. FOXM1 plays a critical role in mediating resistance to genotoxic agents, γ-irradiation and epirubicin 86, 87. Importantly, FOXM1 overexpression is correlated with cisplatin resistance in breast 88 and ovarian cancer 89 as well as docetaxel chemoresistance in gastric cancer 90. Therefore, we predicted that down regulation of FOXM1 by FOXO3 activation would potentially sensitize the resistant cells to drug treatments. In support of this hypothesis, our data demonstrated that UroA or UAS03 treatment significantly upregulated FOXO3 and down regulated FOXM1 in 5FUR cells. Again, knockdown of FOXO3 abrogated UroA or UAS03 mediated regulation of drug transporters. Importantly, knockdown of FOXO3 upregulated FOXM1 in 5FUR cells suggesting a critical role for FOXO3 in regulation of FOXM1-mediated downstream signaling. Here, we showed that treatment with UroA or UAS03 in 5FUR colon cancer cells significantly induced FOXO3 expression and downregulated FOXM1, which in turn is potentially responsible for downregulation of drug transporters. Decreased expression FOXO3 and increased expression of FOXM1, which contribute to increased chemoresistance in cancer has been reversed in co-treatments (5FU+UroA or 5FU+UAS03) suggesting potential translational applications. The direct role of AhR and Nrf2 in UroA or UAS03-mediated activities in 5FU-sensitization mechanisms requires further investigation.

Tumors generated using HCT-116 5FUR cells in NRGS mice are resistant to 5FU treatment reinforcing a 5FUR tumor phenotype in mice. In agreement with *in vitro* results co-treatment (5FU + UroA/UAS03) significantly reduced 5FUR tumor growth in NRGS mice. These mice do not have mature T cells, B cells, or natural killer (NK) cells. These mice offer an excellent model system to examine human cancer models. The molecular analysis of tumors in these mice showed that co-treatment modulated EMT mediators and downregulated drug transporters. Importantly, we observed increased FOXO3 and decreased FOXM1 in these tumors as well, corroborating the *in vitro* studies. As suggested above UroA/UAS03 sensitize 5FUR tumors to 5FU treatment potentially through the FOXO3-FOXM1-drug transporters axis. In future studies, the specific knockout of these regulators *in vivo* would provide better understanding of the mechanisms underlying activity of UroA and UAS03. Since UroA/UAS03 were delivered orally, we still do not know the distribution of these compounds to the tumor site. It will be interesting to determine how these compounds are distributed and reach tumor sites to exhibit such profound anti-tumor activity. Since the immune system is compromised in NRGS mice, we adopted another independent AOM-DSS colon tumor model using C57BL/6 mice to determine the effects of combination therapy in immune sufficient mice. It is evident from data that co-treatment (5FU+UroA/UAS03) effectively reduced tumor numbers and size. Despite the 1 or 2 tumors that appeared in the co-treatments, they are under 1-2 mm in size. It is important to note that mono treatment at the dose (20 mg/kg) and frequency (twice a week) tested in this experiment failed to reduce the tumor burden compared to vehicle. While it is possible that increase in the dose or frequency of mono treatment may reduce the tumor burden that requires further investigation. However, the goal of the present study was to minimize the dose and frequency to mitigate colon tumors, which we achieved in our experimental settings. We selected this dosage based on our experience in chronic DSS-induced colitis. It is worthwhile to test the efficacies at different doses and frequencies in future studies in this model.

Moreover, the immune phenotypes of these mice are very intriguing, where combination therapy significantly reduced splenomegaly and size of lymph nodes as well as systemic inflammatory cytokines. It is well-established that colon cancer patients exhibit increased gut permeability and expression of inflammatory cytokines 91, 92. Since, UroA or UAS03 treatment enhances gut barrier function through upregulating tight junction proteins and reducing inflammation in ulcerative colitis models 39, it is possible that UroA/UAS03 treatment could potentially provide an additional layer of protection in reducing colon cancer burden. In this regard it is pertinent to recall an elegant study from Dr. Egilmez's group, where they showed that enhancing gut barrier integrity by combination treatment with IL-10 and IL-12 sensitizes colon cancer to immunotherapy 93. Authors showed that combination therapy induced a 3-fold increase in tight junction protein levels in the colon and found that IL-10 blocked the detrimental effect of the IL-12-IFNγ axis on barrier function without interfering with its beneficial immunological activity 93. It is possible that increased barrier function by treatment with UroA or UAS03 could result in favorable outcome in eliminating tumors in AOM-DSS models. Numerous reports suggest that gut microbiota and their metabolites play a significant role in regulating outcomes of colon cancer. Our *in vitro* and *in vivo* data suggest that a single microbial metabolite, UroA or UAS03 effectively attenuated tumor burden when combined with 5FU. Future studies are required to delineate mechanisms of these compounds as anti-cancer vs anti-inflammatory and anti-tumor activities of immune response in AOM-DSS tumor models.

We acknowledge that there are many mechanisms responsible for developing 5FU resistance, similarly UroA/UAS03 may regulate 5FU sensitization through various mechanisms other than what have shown in this manuscript. For example, UroA is known to regulate AhR-Nrf2 dependent pathways 39, p53-dependent pathways 94-98 and mitophagy 23. In some instances, activation of Nrf2 was shown to be critical for development of resistance to chemotherapeutic agents 99, 100. Additionally, mitophagy was shown to a play an important role in chemoresistance 101, 102. The *p53* tumor suppressor gene is mutated in almost 50% of human tumors and plays an important role in genotoxic stress and hypoxia 103. We have utilized two cells line HCT-116, wild type for p53 and SW-480 mutant for p53 (E8 codon 273, mutation G to A resulting in Arg to His). In our studies, we observed that treatment with UroA/UAS03 significantly chemosensitized to the 5FU treatment in both cell lines. It was shown that UroA induces prostate cancer cell death in p53-dependent and p-53-independent manner 96. It is possible that UroA may operate similar mechanisms in colon cancer cell lines to induce apoptosis Recently, Giménez-Bastida et al showed that UroA induced p53-dependent cellular senescence in human colon cancer cells, HCT-116, but not in colon cancer lines with p53 mutated or non-tumorigenic cells 97. These studies suggested that long-term UroA-induced senescence-mediated chemoprevention is a p53-dependent manner 97. More in-depth mechanistic studies are required to define the importance of p53 in UroA or UAS03-mediated chemosensitization against 5FU treatment in 5FUR cancers. Therefore, further studies are required to determine the exact role of UroA-Nrf2 and p53 (mutational status) pathways as well as UroA-induced mitophagy in sensitizing 5FUR cancer would provide additional mechanisms for the observed phenotypes. Addressing these outstanding questions would offer better understanding to design the therapeutics to target 5FUR cancers.

**Figure 9:**
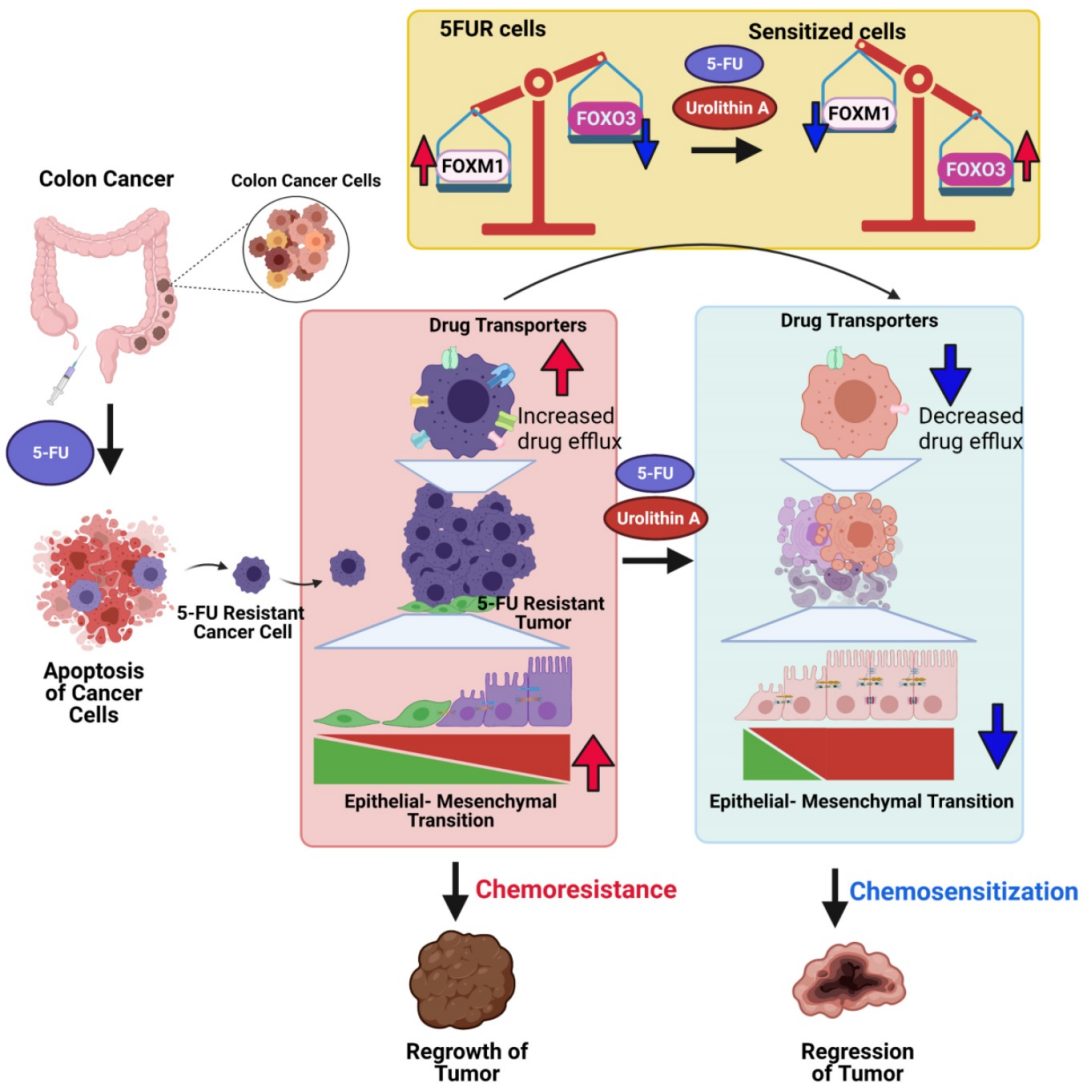
** Potential mechanisms of Urolithin A and its analogue mediated chemosensitization of 5FUR colon cancers.** 5FUR cells escape 5FU treatment and exhibit increased expression of drug transporters, EMT makers with more invasive properties. Increased expression of drug transporters results in increased drug efflux. UroA/UAS03 in combination with 5FU treatment leads to decrease in drug transporters and results decreased drug efflux and increased intracellular drug concentration. Co-treatments reduce EMT markers. UroA/UAS03 mediate chemosensitization through FOXM1-FOXO3 dependent pathway, where co-treatment leads to increase in FOXO3 and decrease FOXM1 expression.

Microbial metabolites particularly the three short-chain fatty acids (SCFAs) butyrate, acetate, and propionate play an immunomodulatory role and induce apoptosis in colon cancer cells 104 and reshape the mucosal immune system by via regulation of colonic adaptive immunity 105, 106. Cancer chemoprevention by microbial metabolites such as polyphenols is also documented both *in vitro* and *in vivo* in the case of colon cancer 107, 108. Previously, we and others showed that treatment with UroA and its analogue UAS03 are not toxic in preclinical models. Recent studies in humans suggested that oral consumption of UroA is not toxic and is approved by FDA as generally recognized as a safe (GRAS) dietary supplement 37. Therefore, direct consumption UroA in combination with 5FU treatment potentially have beneficial outcomes in cancer therapy. In this report, we have shown the novel function of UroA and its analogue in downregulating drug transporters potentially through regulation of the FOXO3-FOXM1 axis in 5FUR colon cancer (Figure 9). We propose that utilization of UroA or its analogues may be safer in the clinic in conjunction with existing 5FU chemotherapy than currently used compounds to overcome 5FU resistance and could potentially overcome 5FUR colon cancer. Therefore, gut microbial metabolites as adjuvant therapeutic along with chemotherapy will enable us to develop novel treatment strategies to overcome drug resistance in colon cancer.

## Supplementary Material

Supplementary figures.

## Figures and Tables

**Table 1:** List of the antibodies and source.

Antibody	Company	Catalog number	Method*	Dilution
Bax	Novus Biologicals	NBP1-28566	WB	1:500
Cleaved Caspase-3	Cell Signaling Technology	9664	IHC	1:100
Cleaved Caspase-9	Cell Signaling Technology	52873	WB	1:1000
Caspase-9	Cell Signaling Technology	9508	WB	1:1000
E cadherin	Cell Signaling Technology	14472	WB IHC	1:1000, 1:100
ZO-1	ProteinTech	A21773-1-AP	WB	1:1000
Snail	Cell Signaling Technology	3879	WB	1:1000
Beta-Catenin	Cell Signaling Technology	8480	WB IHC	1:1000, 1:100
MDR1	Santa Cruz Biotechnologies	sc -55510	WB IHC	1:1000, 1:100
BCRP	R&D Systems	MAB995	WB	1:2000
MRP2	Santa Cruz Biotechnologies	sc-71603	WB	1:200
FOXO3	Novus Biologicals	NB100-614	WB	1:1000
FOXM1	Santa Cruz Biotechnologies	sc-376471	WB	1:500
MRP7	Invitrogen	PA575402	WB	1:250
HRP-Conjugated GAPDH	ProteinTech	HRP-60004	WB	
HRP-Conjugated Beta Actin	ProteinTech	HRP-60008	WB	1:5000
Goat anti-mouse IgG (H+L), HRP conjugate	ProteinTech	SA00001-1	WB	1:5000
Goat anti-rabbit IgG (H+L), HRP conjugate	ProteinTech	SA00001-2	WB	1:5000
IgG2a Cross-Adsorbed Goat anti-Mouse, Alexa Fluor® 594, Invitrogen™	Invitrogen	A21135	IHC	1:200
IgG (H+L) Cross-Adsorbed Goat anti-Rabbit, Alexa Fluor® 488, Invitrogen™	Invitrogen	A11008	IHC	1:200

*WB: Western blot; IHC: Immunohistochemistry
