# Supplementary material for: Microbial metabolite restricts 5-fluorouracil-resistant colonic tumor progression by sensitizing drug transporters via regulation of FOXO3-FOXM1 axis
Source: Theranostics. 2022 Jul 18;12(12):5574–95. doi: 10.7150/thno.70754 (PMC9330515; doi:10.7150/thno.70754)
Supplement: Supplementary file 1 — Supplementary figures. [file thnov12p5574s1.pdf]

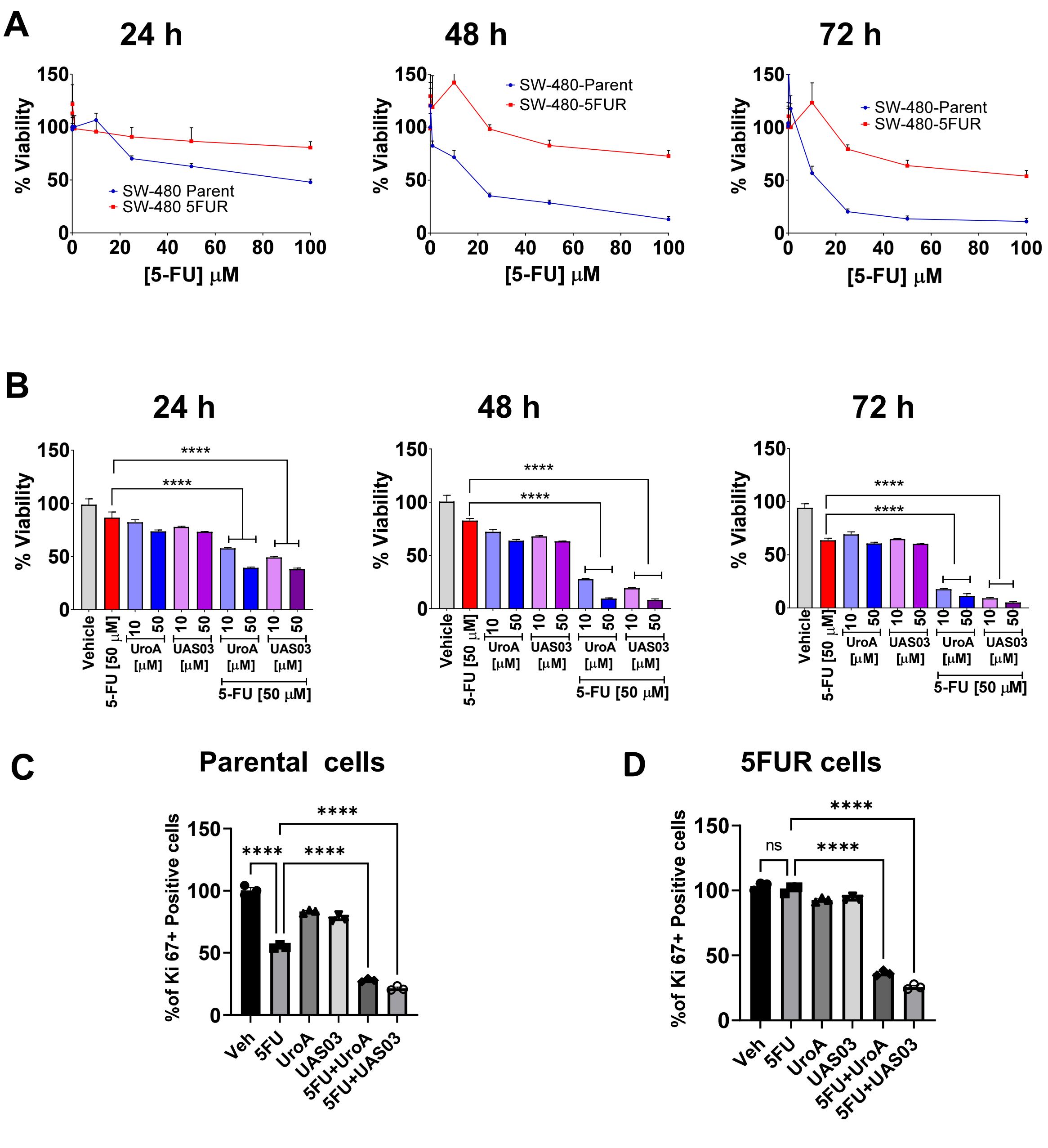

Figure S1. UroA and UAS03 chemosensitize the SW480-5FUR colon cancer cells. A. SW480-parental or SW480-FUR cells (7,000 cells/well) were plated in 96-well plate and grown for overnight. Cells were treated with indicated compounds in dose dependent manner for 24, 48, 72 h. The cell proliferation was measured using standard MTT assay. **B.** Cell viability (SW480 FUR) co-treatments as indicated was calculated against DMSO (0.1%) as 100% Cell proliferation was evaluated by Ki67 positive cells using flowcytometry methods in SW480 parental (**C**) and 5FUR cells (**D**). Data are represented as mean ± S.E.M. of three different experiments. ns: non-significant; ****$P < 0.0001$.

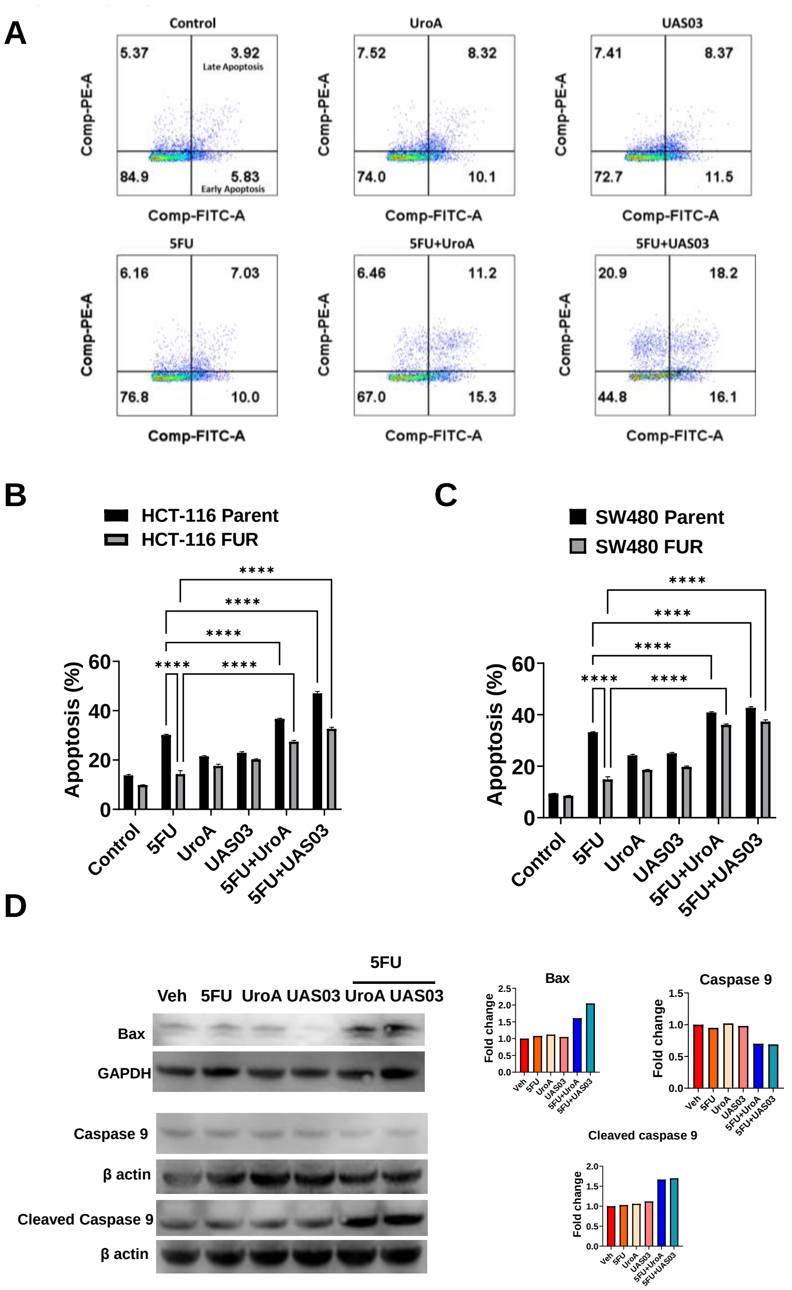

Figure S2. Co-treatment with UroA or UAS03 with 5FU promote apoptosis of 5FUR cells. A. Representative flowcytometry of Annexin V-FITC and propidium iodide-PE of HCT-116 5FUR cells treated with UroA/UAS03/5FU in the presence or absence of 5FU. **B-C.** HCT-116-parental and HCT-116-FUR **(B)** or SW-480 parental and SW480-FUR **(C)** cells were treated with either 5FU (50 µM), UroA (50µM), UAS03 (50µM) or 5FU (50 µM) in combination of UroA (50µM) or UAS03 (50µM) for 24 h. Cells were stained with annexin V-FITC and propidium iodide (PI) and analyzed for apoptosis using flow cytometry. Data are represented as mean ± S.E.M. of three different experiments. Statistics performed one way ANOVA using Graph Prism Software. $^{****}P < 0.0001$. **D.** HCT-116 5FUR cells treated with either 5FU (50 µM), UroA (50µM), UAS03 (50µM) or 5FU (50 µM) in combination of UroA (50µM) or UAS03 (50µM) for 24 h and cell lysates prepared using RIPA plus buffer. The cell lysates were loaded on SDS-PAGE and the expression of indicated apoptosis markers was analyzed by Western blot analysis.

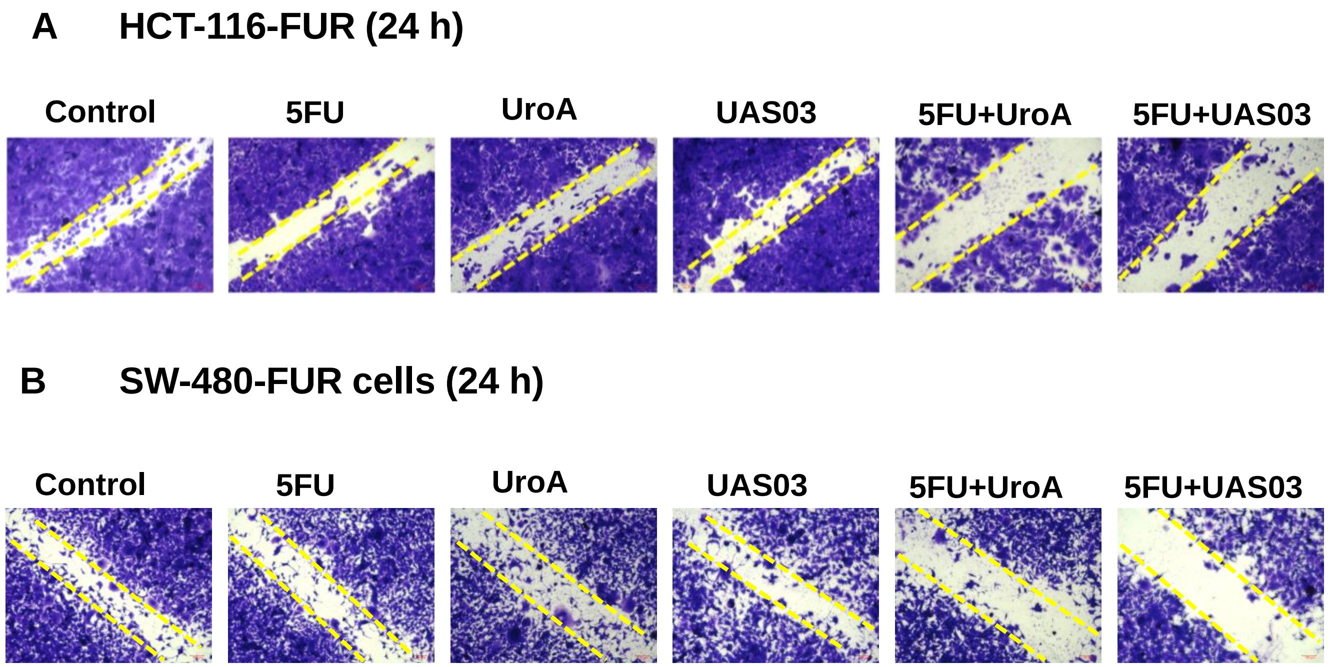

Figure S3. UroA and UAS03 co-treatment with 5 FU reduce cell migration in 'scratch assay'. HCCT-116-FUR **(A)** SW480-FUR cells (1X10$^{5/}$well) **(B)** were plated in 12 well plate and allowed them to form monolayer. 'Scratch' was made using sterile p200 pipette tip and washed cells. The cells were treated with vehicle (0.1% DMSO) or UroA or UAS03 (50 µM) in the presence of 5 FU (50 µM). After 24 h, the cells were stained with crystal violet and images were captured using microscope.

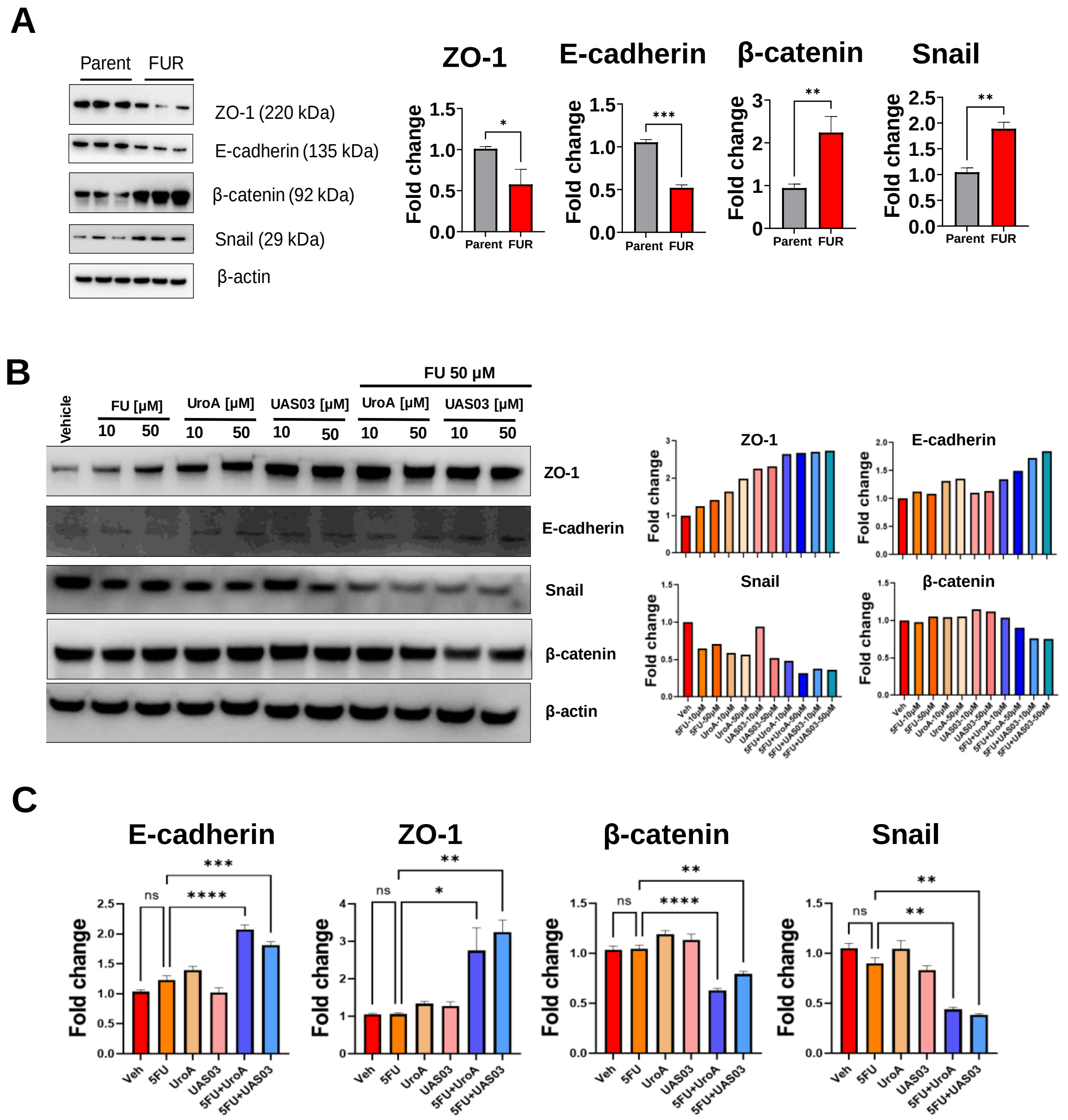

Figure S4. 5FU modulates EMT markers upon co-treatment with UroA/UAS03 in SW480 parental and SW480-5FUR cells. A. Western blot analysis of EMT makers in SW480 parental and SW480-5FUR cells. **B.** Western blot analysis of EMT makers in SW480-FUR cells treated with 5-FU in the presence of UroA or UAS03 at indicated doses. **C.** Total RNA was isolated from SW480-FUR cells and analyzed for the expression of E-cadherin, ZO-1, β-catenin and Snail. The fold changes in mRNA levels were determined by RT PCR method. Error bars, ±SEM. Statistics performed one way ANOVA using Graph Prism Software. ns: non-significant, $*p < 0.05$, $**p < 0.01$, $***p < 0.001$

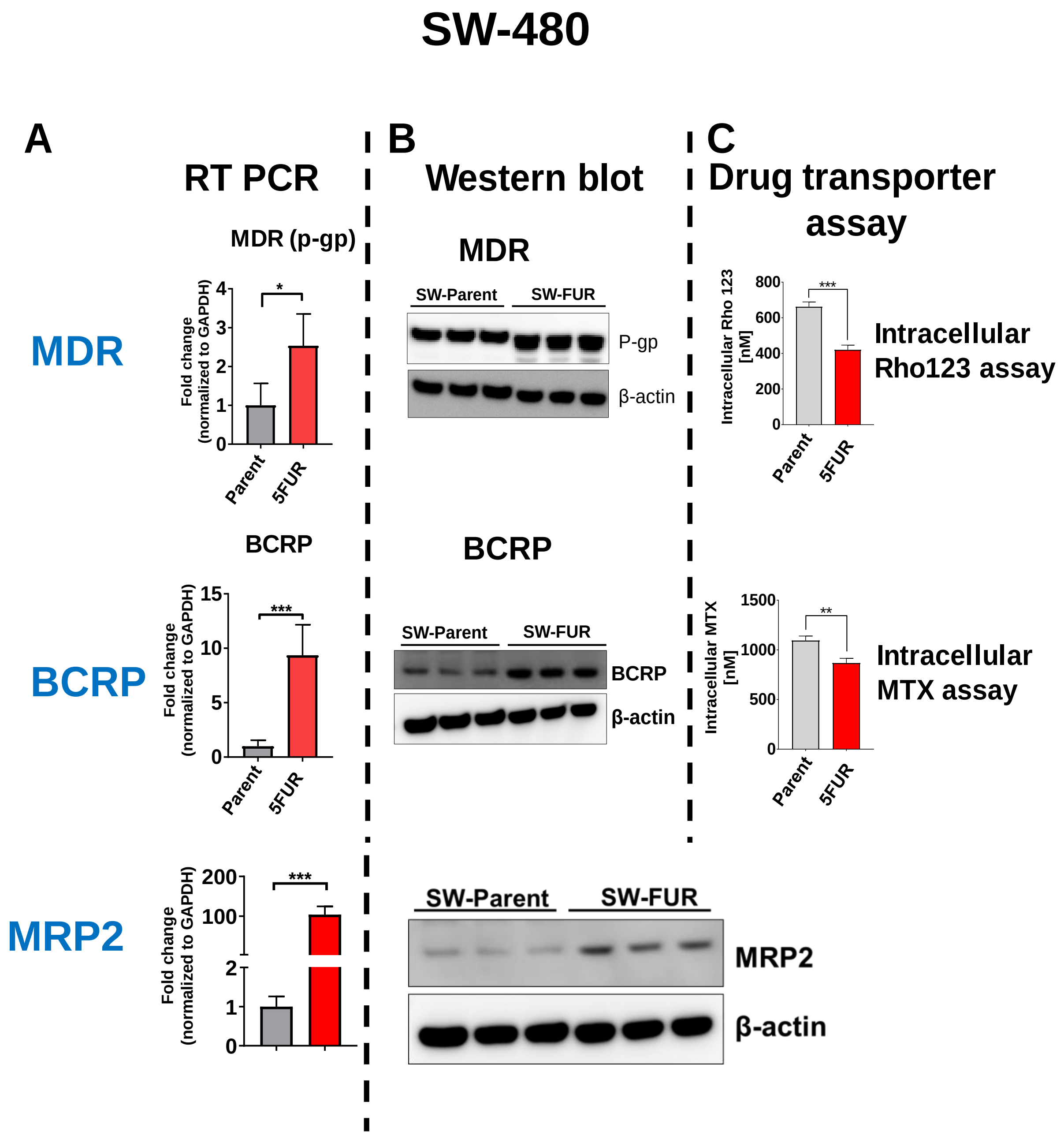

Figure S5. Increased levels of drug transporters in SW480-FUR cells. A. Total RNA was isolated from parental SW480 and SW480-FUR cells and analyzed expression of MDR, BCRP and MRP2. **B.** Cell lysates of parental SW480 and SW480-FUR cells were subjected to Western blot analysis for the indicated proteins. **C.** Drug transporter assays for MDR and BCRP were performed using Rho123 and MTX Intracellular levels of these drugs were measured. Statistics performed using unpaired t-test. ns: non-significant, *$p < 0.05$, **$p < 0.01$, ***$p < 0.001$**.** Error bars indicate ±SEM

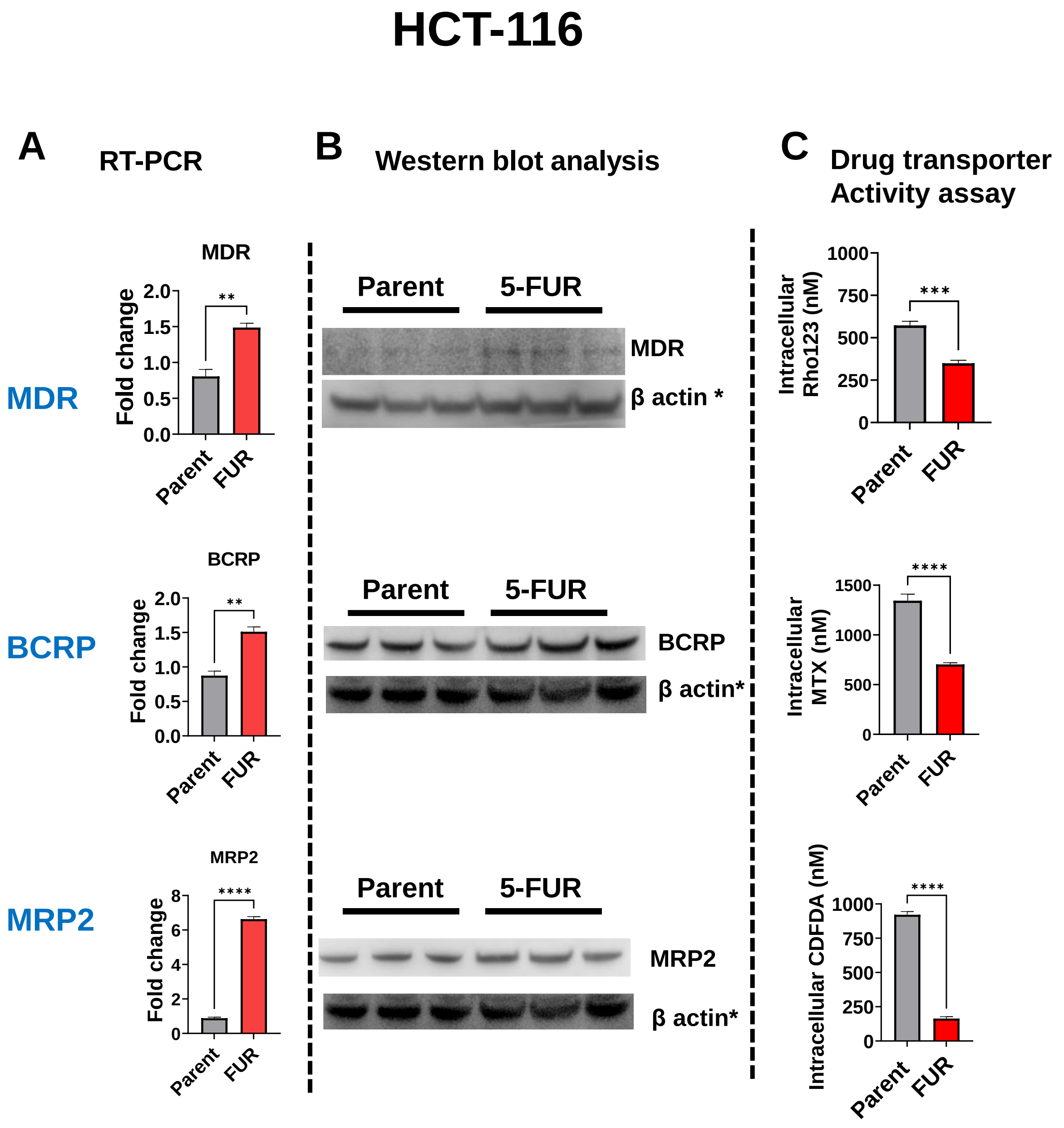

Figure S6. Increased levels of drug transporters in HCT-116 FUR cells. A. Total RNA was isolated from parental HCT-116 and HCT-116-FUR cells and analyzed expression of MDR, BCRP and MRP2 **B.** Cell lysates of parental HCT-116 and HCT-116-FUR cells were subjected to Western blot analysis for the indicated proteins. **C.** Drug transporter assays for MDR and BCRP were performed using Rho123, MTX and CDFDA Intracellular levels of these drugs were measured. Statistics performed using unpaired t-test. ns: non-significant, *$p < 0.05$, **$p < 0.01$, ***$p < 0.001$**.** Error bars indicate ±SEM

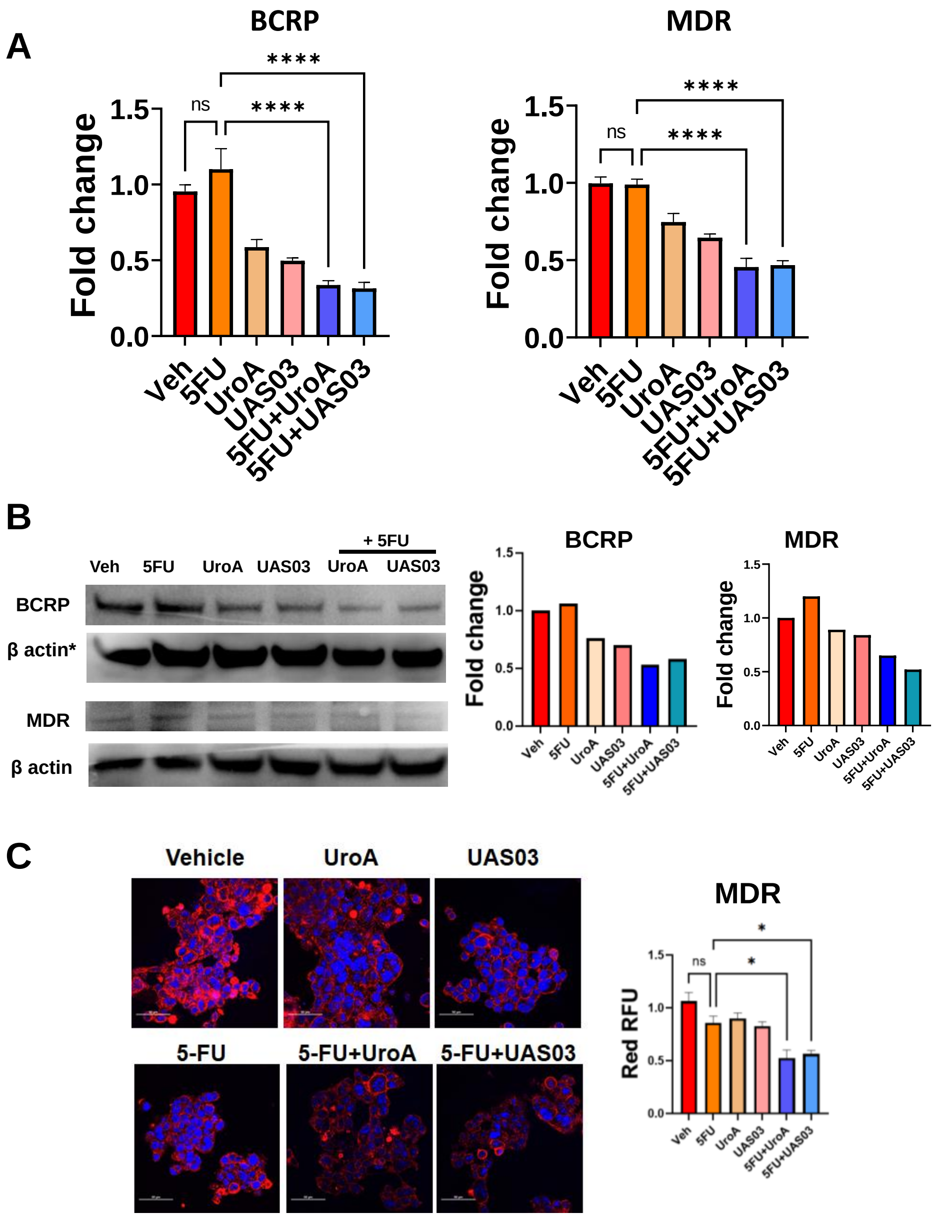

Figure S7. Co-treatment with UroA/UAS03 and 5FU reduce expression of drug transporters **A.** Total RNA was isolated from HCT116-FUR cells treated with vehicle or 5FU (50 µM) in combination with UroA or UAS03 (50 µM) for 24 h. mRNA levels were determined by real time PCR using SyBR green method. **B.** Cell lysates of HCT-116-FUR cells treated with vehicle or 5FU (50 µM) in combination with UroA or UAS03 (50 µM) for 24 h were subjected to Western blot analysis for the indicated proteins. **C.** HCT-116-FUR cells were grown on 8 well chamber slide and treated with either 5FU (50 µM), UroA (50µM), UAS03 (50µM) or 5FU (50 µM) in combination of UroA (50µM) or UAS03 (50µM) for 24 h. The cells were stained with anti-MDR antibody followed by secondary antibody tagged with Alexa-594. Nucleus was stained using DAPI. The confocal images were captured. Scale bars indicate 50 µm. Error bars, ±SEM. Statistics performed one way ANOVA using Graph Prism Software. ns: non-significant, *$p < 0.05$, **$p < 0.01$, ***$p < 0.001$

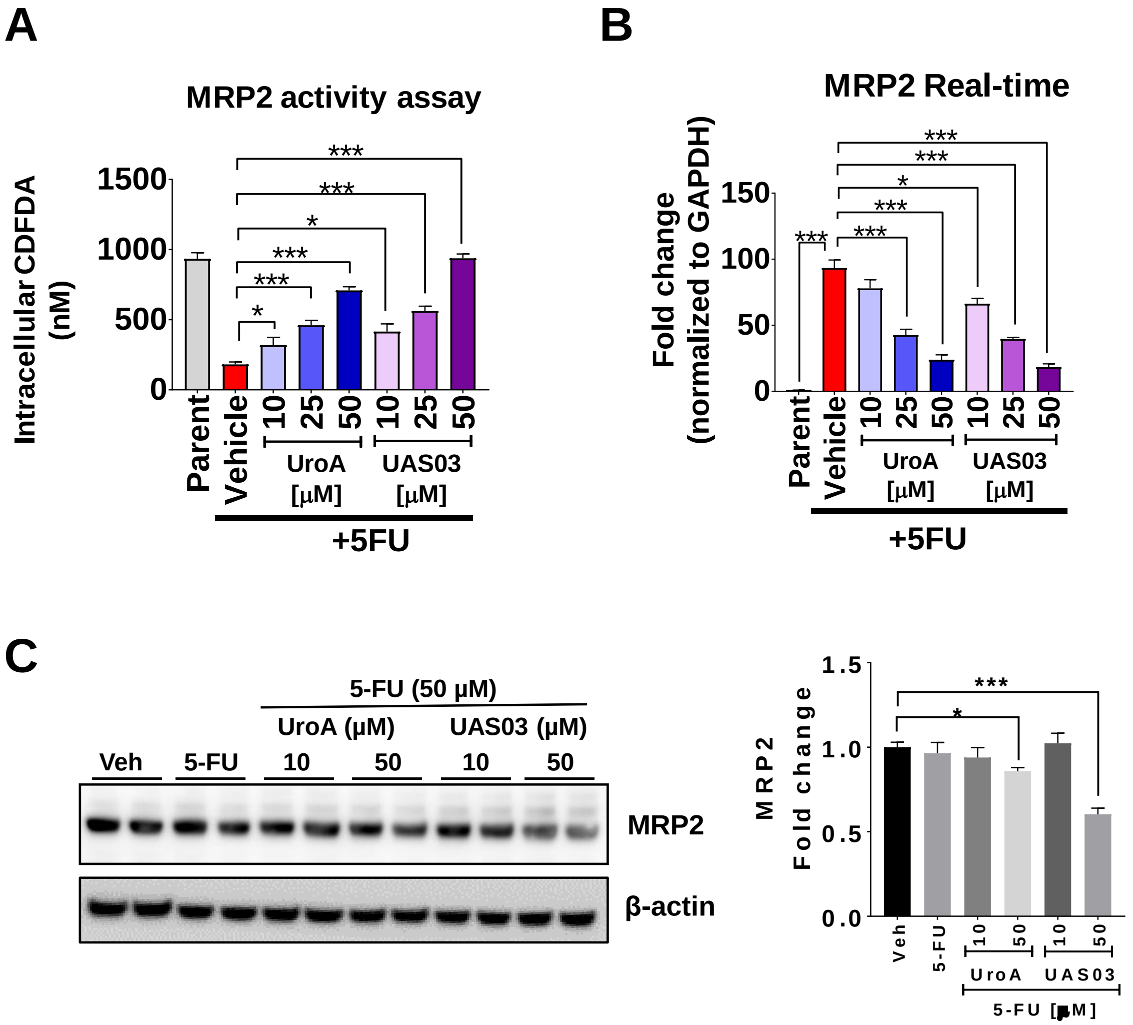

Figure S8. Treatment with UroA and UAS03 reduce expression and activity of MRP2. A. Parental SW480 and SW480-FUR cells were used evaluate MRP2 efflux of CDFDA. MRP2 transporter assay was performed using CDCFDA substrate and measured intracellular CDCFDA after incubation 1 h in the presence of Vehicle (0.1% DMSO) or UroA or UAS03 and 5FU (50 µM) **B.** Parental SW480 and SW480-FUR cells were treated with vehicle (0.1% DMSO) or UroA or UAS03 (10, 25, 50 µM) and 5FU (50 µM) for 24 h. Total RNA was isolated and mRNA levels of MRP2 were determined by realtime PCR. **C.** SW480-FUR cells treated with UroA or UAS03 in the presence of 5 FU (50 µM) for 24 h. MRP2 protein levels were measured by Western blots and quantified fold change. Statistics performed one way ANOVA using Graph Prism Software. *$p < 0.05$, **$p < 0.01$, ***$p < 0.001$

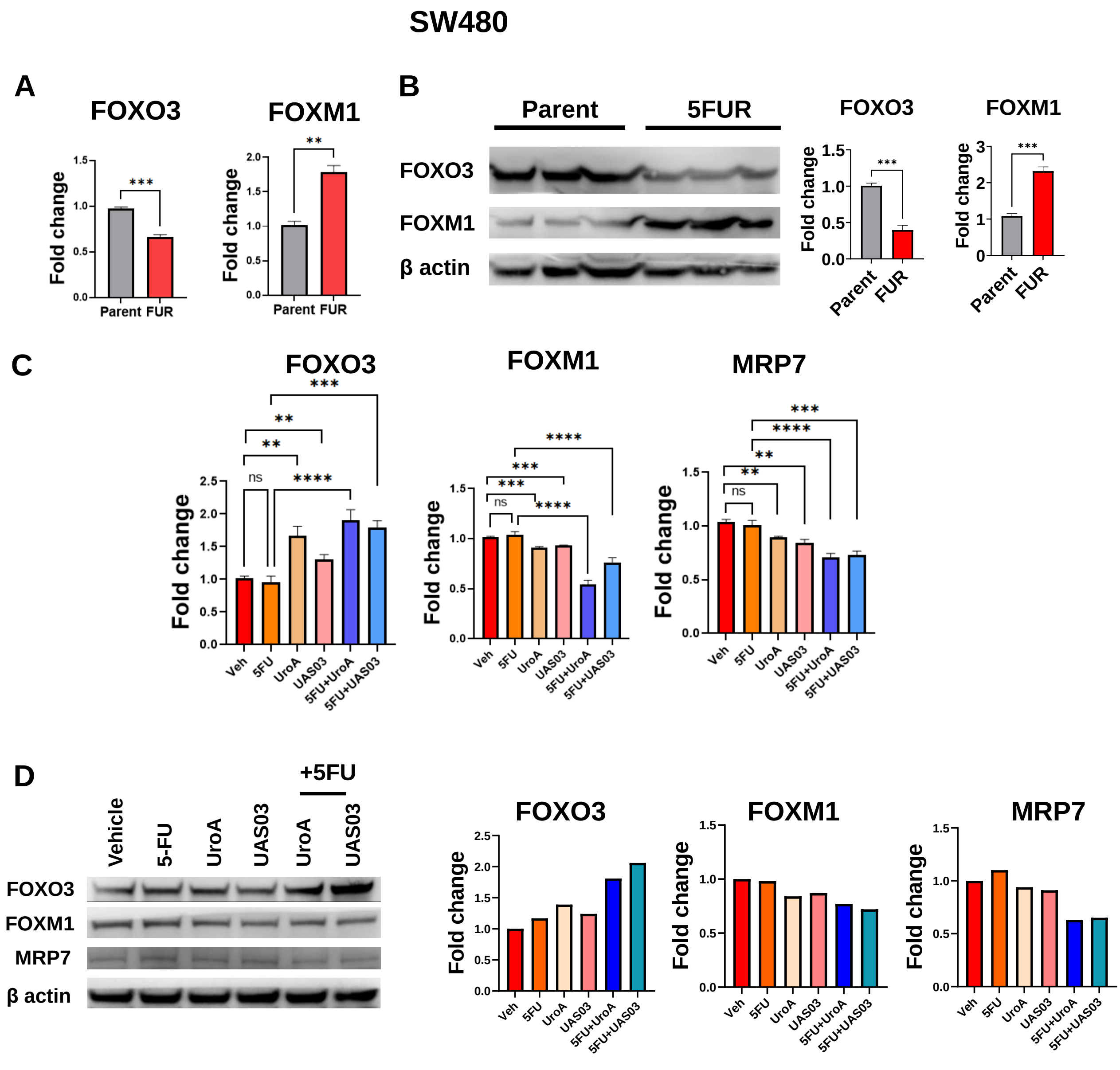

Figure S9. Treatment with UroA/UAS03 regulates the FOXO3-FOXM1 axis. A. Total RNA was isolated from parental SW480 and SW480-FUR cells and analyzed the mRNA expression of FOXO3 and FOXM1.The fold changes in mRNA levels were determined by RT PCR method**. B.** Western blot analysis of FOXO3 and FOXM1 in parental SW480 and SW480-FUR cells. **C.** Total RNA was isolated from SW480-FUR cells treated with either 5FU (50 µM) or UroA (50 µM) or UAS03 (50 µM) or in combination for 24 h. The mRNA expression of FOXO3, FOXM1 and MRP7 was determined by SyBR RT PCR. The fold changes in mRNA levels were represented. **D.** SW480-FUR cells were treated with either 5FU (50 µM) or UroA (50µM) or UAS03 (50µM) or in combination for 24 h. Western blot analysis of FOXO3, FOXM1 and MRP7 is shown. Statistics performed one-way ANOVA using Graph Prism Software. Error bars, ±SEM. **p < 0.01, ***p < 0.001.

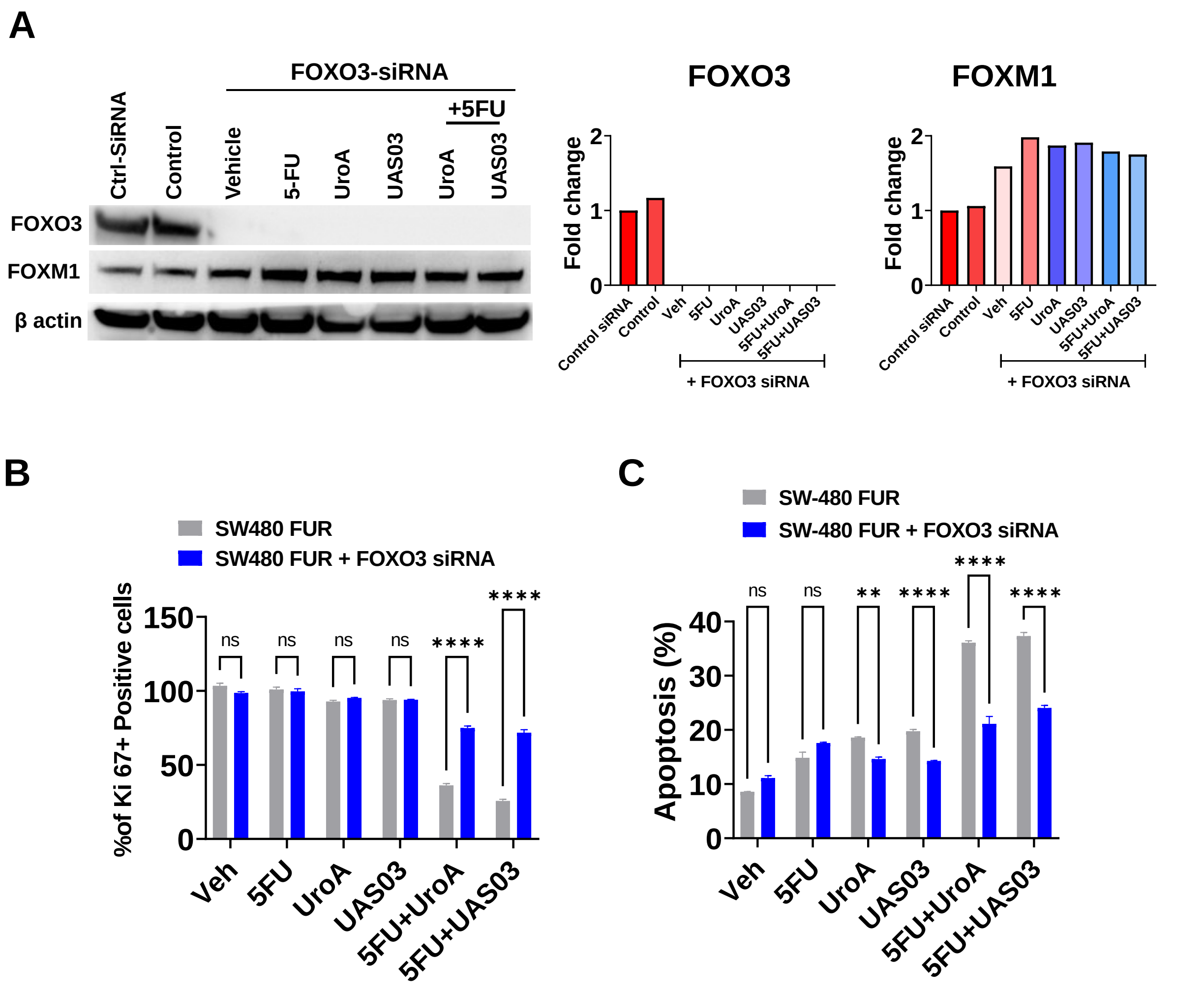

Figure S10. Knockdown of FOXO3 abrogated UroA/UAS03-mediated chemosensitization activities. A. FOXO3 was knockdown in SW480-5FUR cells using FOXO3-siRNA. Expression of FOXO3 was confirmed by Western blot. These cells were treated with either 5FU (50 µM) or UroA (50µM) or UAS03 (50µM) or in combination for 24 h, and expressions of FOXM1 and FOXO3 were determined by Western blots. Cell proliferation by Ki67 staining **(B)** and apoptosis by Annexin V and PI staining were measured using flow cytometry methods (**C**) Statistics performed one-way ANOVA using Graph Prism Software. Error bars, ±SEM. $^{**}p < 0.01$, $^{***}p < 0.001$.

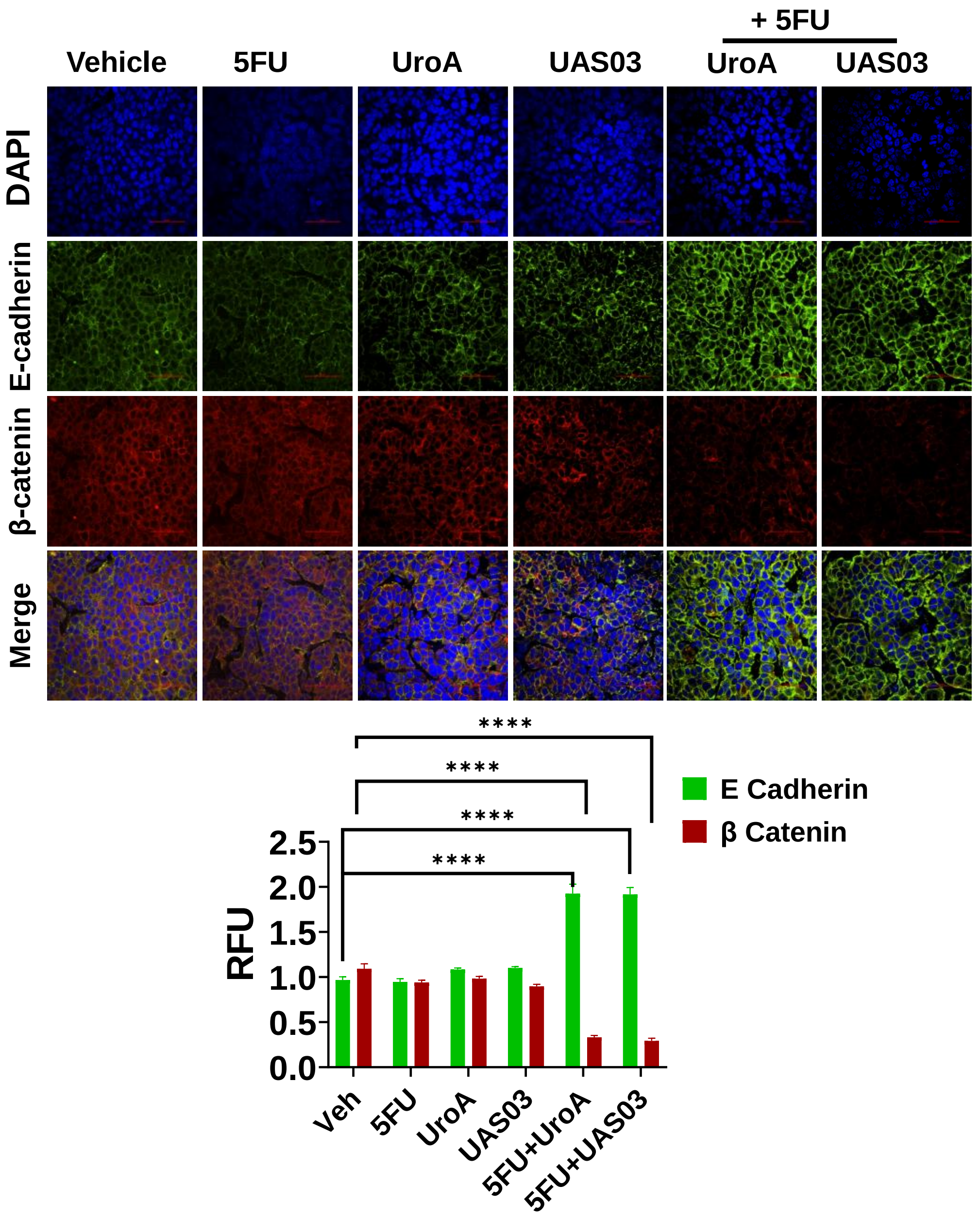

Figure S11. 5FU modulates EMT markers upon co-treatment with UroA/UAS03 in 5FUR tumors. Tumor sections were stained with rabbit anti E-cadherin and mouse anti-β Catenin, followed by secondary antibody tagged with anti-rabbit Alexa 488 and anti-mouse Alexa-594. Nucleus was stained using DAPI. The representative florescence images were captured using Nikon A1R confocal microscope. Scale bars indicate 50 µm. The fluorescence intensity ($n = 15–20$ cell membrane regions) was measured. Statistics performed using one-way ANOVA in GraphPad Prism software. Error bar, mean ± SEM,****$p < 0.001$.

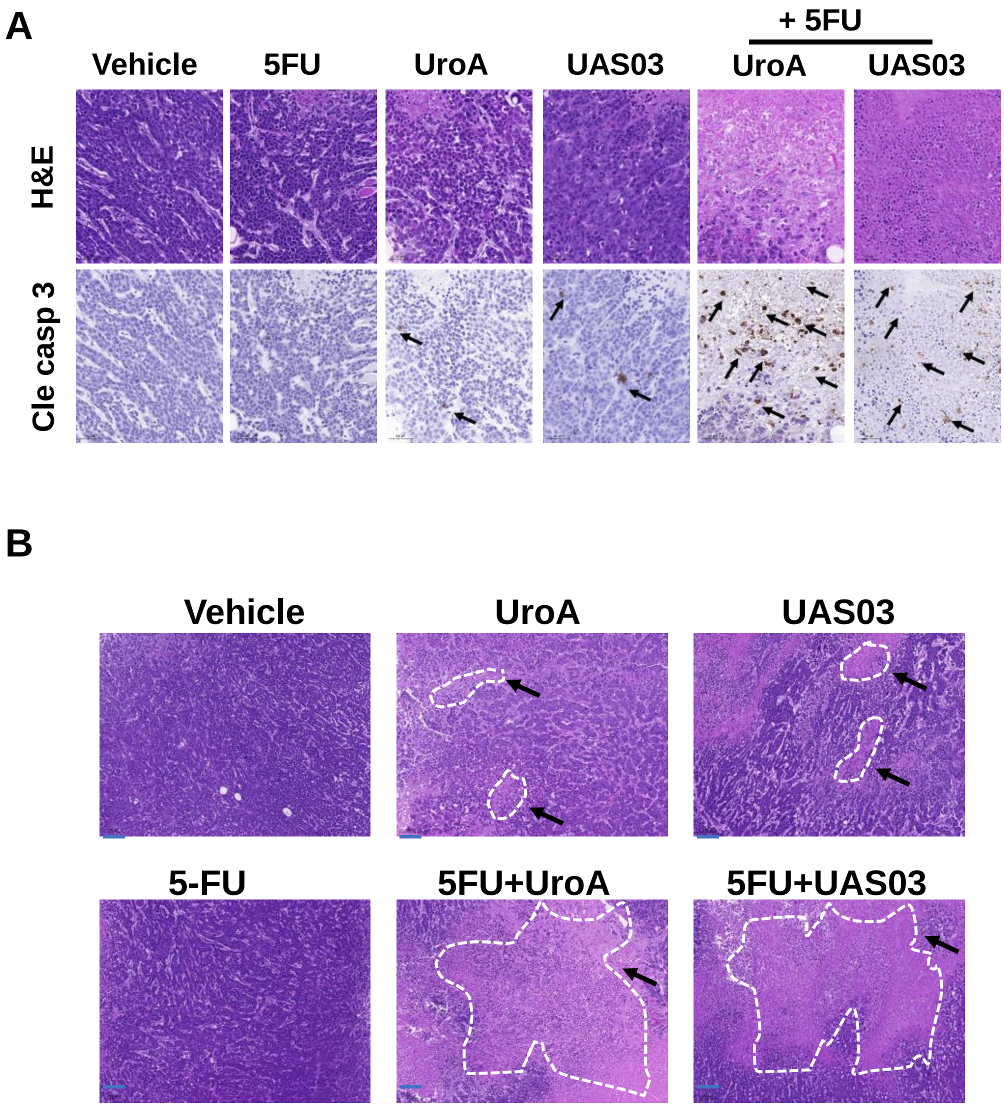

Figure S12. UroA/UAS03 treatment in combination with 5FU reduced tumors and induced apoptosis. (A) Representative H&E staining images of the tumor sections (Upper panel). In H&E staining, nuclei were stained blue, the cytoplasm and extracellular matrix were stained pinkish. The tumor sections were stained for cleaved caspase 3 (lower panel) by IHC staining. Black arrows indicate the presence of cleaved caspase 3. Images were captured at 200 x magnification. Embedded scale bar represents 50 µm. **(B)** In representative H&E-stained images of the tumor sections, the white dotted lines show the tumor necrosis region. The blue scale bar represents 100 µm.

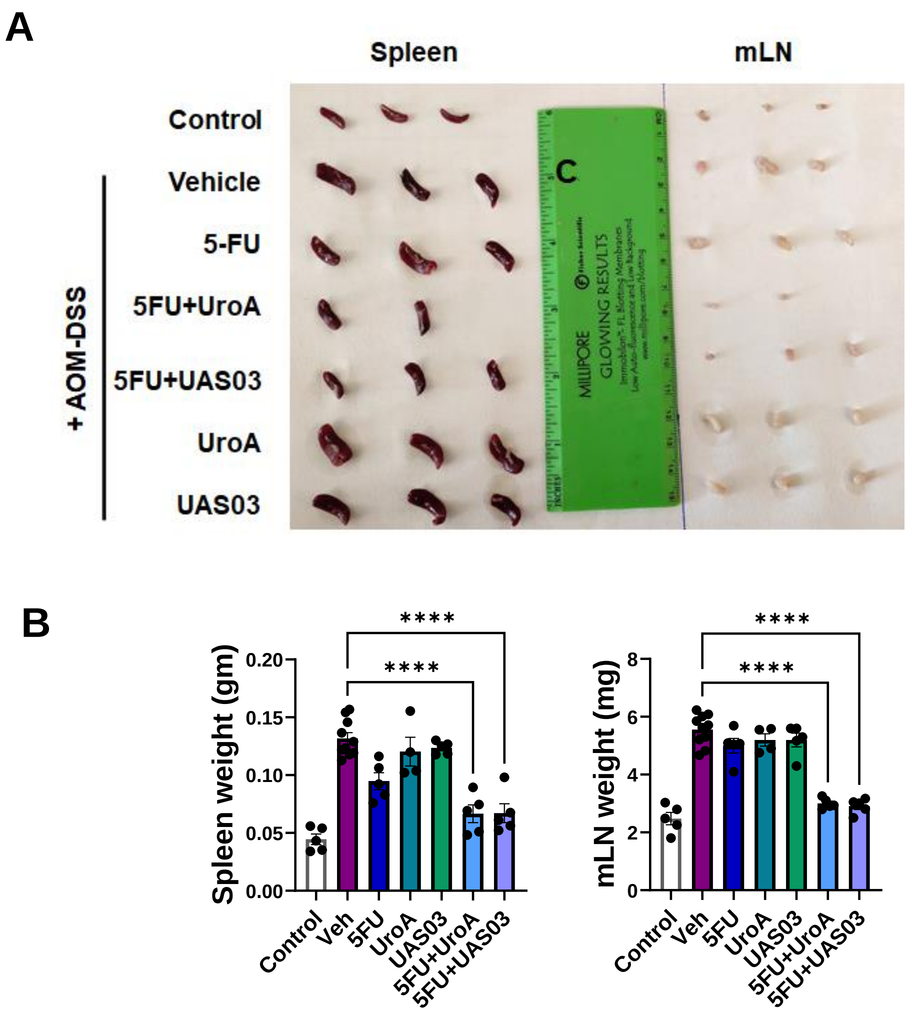

Figure S13. 5FU in combination with UroA or UAS03 treatment protects against AOM-DSS induced splenomegaly and swelling of mesenteric lymph nodes (mLN). A. Representative images of spleen and mLN. **B.** Bar diagram representing total weights of spleen and mLN. Error bars, ±SEM, Statistics performed one way ANOVA using Graph Prism Software. ****$p < 0.0001$.

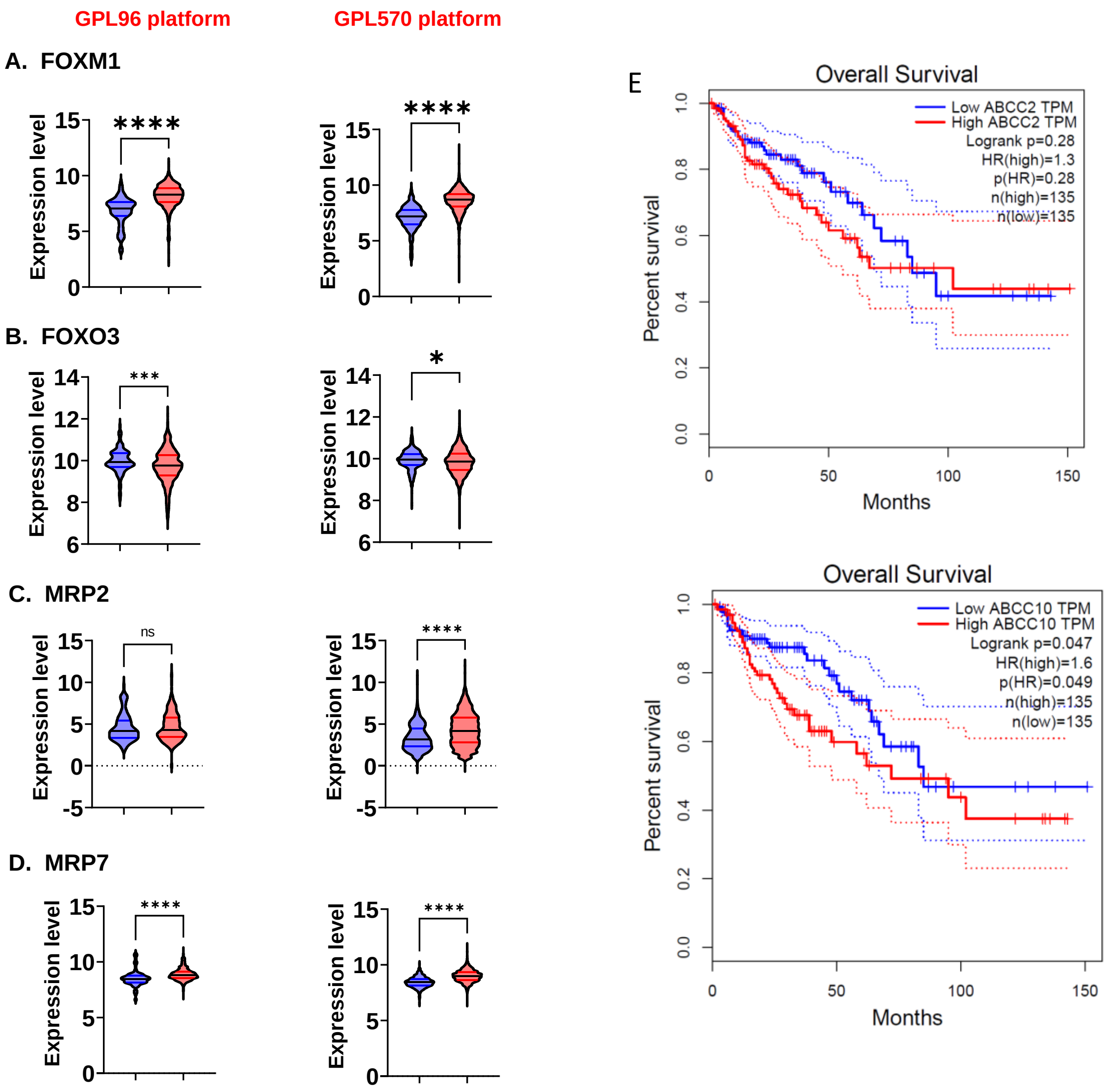

Figure S14. Expression analysis of indicated genes in normal and colon cancer human subjects. A-D. Expression of FOXM1, FOXO3, MRP2 and MRP7 were obtained from Gene Expression database of Normal and Tumor tissues 2 (GENT2) (http://gent2.appex.kr). In this analysis, authors collected data from the GEO public repository using two platforms, U133Plus2 (GPL570) and U133A (GPL96). The above analysis contains normal colon tissues (n = 397 in GPL570 set; n = 127 in GPL96 set) and colon cancer tissues (n = 3775 in GPL570 set; n = 1112 in GPL96 set) samples. Statistics performed using two-sample t-test. *p < 0.05; ****p < 0.0001. **E.** Survival data from resource 'GEPIA: a web server for cancer and normal gene expression profiling and interactive analyses' (http://gepia.cancer-pku.cn/). Authors analyzed colon tumors (T) (n = 275) and normal colon tissues (N) (n = 349) and we mined the data for MRP2 (ABCC2) and MRP7 (ABCC10) genes.
